# Practical Synthesis of 2-Iodosobenzoic Acid (IBA) without Contamination by Hazardous 2-Iodoxybenzoic Acid (IBX) under Mild Conditions

**DOI:** 10.3390/molecules26071897

**Published:** 2021-03-27

**Authors:** Hideyasu China, Nami Kageyama, Hotaka Yatabe, Naoko Takenaga, Toshifumi Dohi

**Affiliations:** 1 Department of Medical Bioscience, Nagahama Institute of Bio-Science and Technology, 1266, Tamuracho Nagahama-shi, Shiga 526-0829, Japan; 2 College of Pharmaceutical Sciences, Ritsumeikan University, 1-1-1 Nojihigashi, Kusatsu, Shiga 525-8577, Japan; ph0093ei@ed.ritsumei.ac.jp (N.K.); ph0090ir@ed.ritsumei.ac.jp (H.Y.); 3 Faculty of Pharmacy, Meijo University, 150 Yagotoyama, Tempaku-ku, Nagoya 468-8503, Japan; ntakenag@meijo-u.ac.jp

**Keywords:** cyclic organoiodine(III) compounds, Oxone^®^, water, solvolytic functionalization, mild condition, metal-free, 2-iodosobenzoic acid

## Abstract

We report a convenient and practical method for the preparation of nonexplosive cyclic hypervalent iodine(III) oxidants as efficient organocatalysts and reagents for various reactions using Oxone^®^ in aqueous solution under mild conditions at room temperature. The thus obtained 2-iodosobenzoic acids (IBAs) could be used as precursors of other cyclic organoiodine(III) derivatives by the solvolytic derivatization of the hydroxy group under mild conditions of 80 °C or lower temperature. These sequential procedures are highly reliable to selectively afford cyclic hypervalent iodine compounds in excellent yields without contamination by hazardous pentavalent iodine(III) compound.

## 1. Introduction

**Figure 1:**
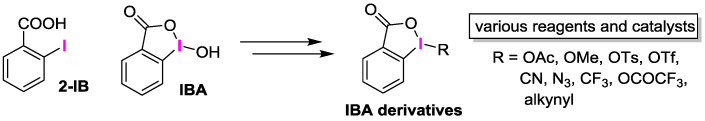
2-Iodosobenzoic acid (IBA) and its precursor 2-iodobenzoic acid (2-IB).

Cyclic hypervalent iodine reagents, such as 2-iodosobenzoic acid (IBA) and 2-iodoxybenzoic acid (IBX) are nonmetallic green oxidants with excellent recyclability [1,2,3]. IBA and IBX can be regenerated from 2-iodobenzoic acid (2-IB) without requiring an external ligand except for water in this reoxidation step. This is because the carboxy group adjacent to the iodine atom serves as an endogenous ligand. Recently, IBA, a representative trivalent cyclic hypervalent iodine oxidant, has been used as a catalyst and reagent in various reactions, i.e., decarboxylative alkynylation [4,5], decarboxylative acylarylation [6], oxyalkenylation [7], oxyarylation [8], oxidative C–H arylation [9], C–H hydroxylation [10], C-H oxidation [11,12], ring-opening hydrazination [13], and asymmetric intramolecular α-cyclopropanation [14]. IBA derivatives containing OAc [15,16,17,18,19,20,21,22,23,24,25,26,27,28], OMe [29,30,31,32], OTs [33,34,35], OTf [36,37], Cl [38,39,40,41,42], F [43,44,45], CN [46], N_3_ [47,48,49,50,51,52,53,54], CF_3_ [55,56], OCOCF_3_ [57], alkynyl [58,59,60,61,62] ligands instead of the hydroxy group have also found application in various reactions (Figure 1).

**Figure 2:**
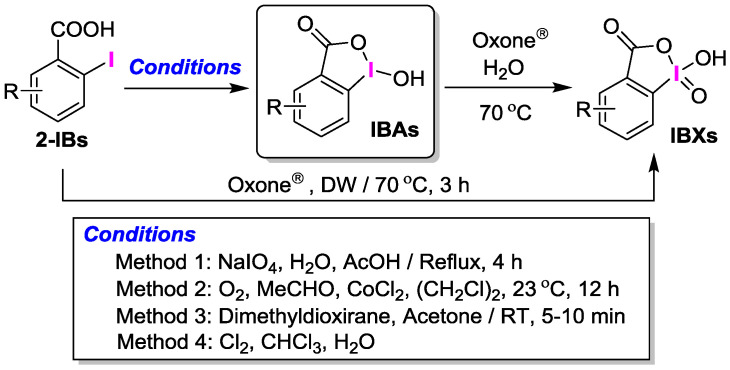
Synthesis of 2-iodosobenzoic acids (IBAs) and 2-iodoxybenzoic acids (IBXs) from 2-iodobenzoic acids (2-IBs).

Although IBAs can be prepared from 2-IBs by existing methods (Figure 2) [63,64,65,66], the development of a safer and more efficient method for their synthesis is highly desirable. As shown in Figure 2, IBAs can be further oxidized to pentavalent cyclic hypervalent IBXs [67], which need to be prevented for the preparation of IBAs [68,69,70], mainly due to the explosive nature of IBXs on heating and impact, while IBXs are useful in some small-scale reactions [71,72,73,74,75,76]. Thus, contamination by IBX in the IBA products should be avoided for long-term safe storage or large-scale use.

In recent decades, many reactions using Oxone^®^, which is an inexpensive and commercially available stable triple salt (2KHSO_5_/KHSO_4_/K_2_SO_4_), have been developed for practical synthetic purposes [77]. In particular, the use of Oxone^®^ as a re-oxidant for pentavalent hypervalent iodine reagents is drawing attention for catalytic oxidation reactions [78,79,80,81,82,83,84,85,86,87,88,89,90,91,92]. The reaction systems for alcohol oxidations [78,79,80,81,82,83,84] involving in situ generated active hypervalent iodine(V) species are optimized on the basis of the preparative conditions of IBX from 2-IB at 70 °C [93]. Meanwhile, oxidative lactonizations from modified 2-IBs using Oxone^®^ occur at room temperature [85,86,87]. In this context, the generation of nonexplosive trivalent cyclic hypervalent iodines, i.e., IBA and its analogs, using Oxone^®^ can be expected to provide a convenient and safe synthetic procedure; however, to best of our knowledge, the selective preparation of IBAs using Oxone^®^ has not been reported so far.

Recently, we reported that IBAs generated in a reaction system containing 2-IB and Oxone^®^ play a catalytic role in the selective oxidation of alkoxybenzenes to *p*-quinones [94]. This resulted in the development of the practical method herein reported for the selective preparation of IBAs under mild conditions.

## 2. Results and Discussion

### 2.1. Selective Synthesis of IBA and Its Analogs

**Figure 3:**
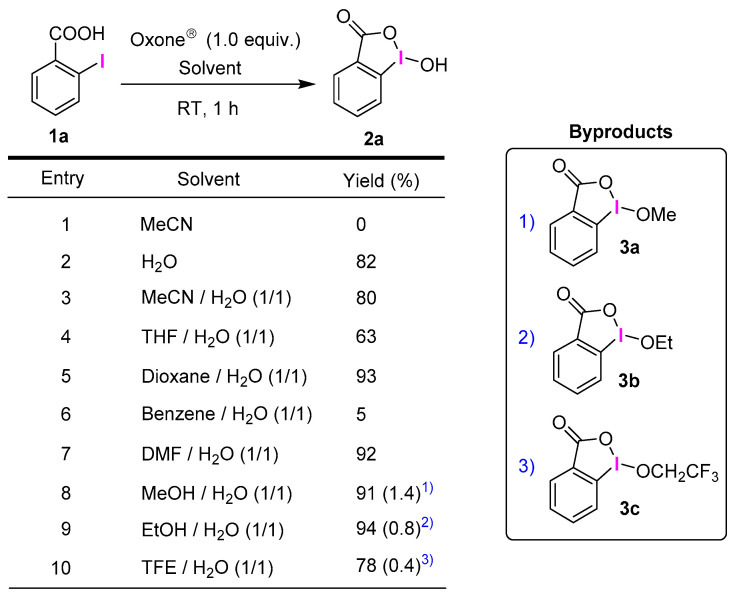
Solvent effect on the selective synthesis of IBA **2a** from 2-IB **1a** using Oxone^®^.

We started our investigation on the selective preparation of IBAs by evaluating the solvent effects on the oxidation of 2-IB **1a** using 1.0 equivalent of Oxone^®^ to obtain IBA **2a** as a representative compound, and the results are summarized in Figure 3. First, the reaction in water led to the successful production of IBA **2a** in 82% yield (Figure 3, entry 2), whereas IBA **2a** was not produced in organic solvent in the absence of water (no reaction because Oxone^®^ was not dissolved) (see entry 1). This result indicates that water plays an essential role in the formation of IBA. Therefore, we assumed that an aqueous system similar to the selective formation of *p*-quinone from alkoxybenzenes catalyzed by 2-IB **1a** with Oxone^®^ [94] could be suitable for the present reaction. We then investigated in detail the effect of a series of organic solvents on the aqueous preparation of IBA **2a** using Oxone^®^.

Various water-miscible organic solvents were investigated to dissolve 2-IB **1a** in this reaction. The preparation of IBA **2a** using acetonitrile (MeCN) in aqueous condition (Figure 3, entry 3) was similar to that performed in the absence of organic solvents (Figure 3, entry 2). Tetrahydrofuran (THF), dioxane, benzene and *N*,*N*-dimethylformamide (DMF) were also examined, finding that the use of highly polar dioxane and DMF led to excellent yields of IBA **2a** (Figure 3, entries 5 and 7), whereas benzene, the least polar solvent among these aprotic solvents, significantly reduced the yield of the desired product (Figure 3, entry 6). The reason for this very low yield IBA formation was interpreted as being due to that benzene forms a two-phase system and interferes with the dissolution of 2-IB into water. Protic solvents such as MeOH, EtOH and 2,2,2-trifuoroethanol (TFE) gave IBA **2a** in high yields; however, they also worked as a ligand for IBA, causing the formation of very small amounts of ligand-exchanged byproducts **3a**–**c** (Figure 3, entries 8–10). The white solid IBA **2a** obtained after the water and acetone washings did not contain any other byproducts. Although this result indicated that protic organic solvents were not suitable for the selective preparation of IBAs, it also revealed that the IBA hydroxyl group could undergo substitution reactions under mild conditions (vide infra). The yields indicated in Figure 3 are almost equal to the conversion of 2-IB **1a**.

**Figure 4:**
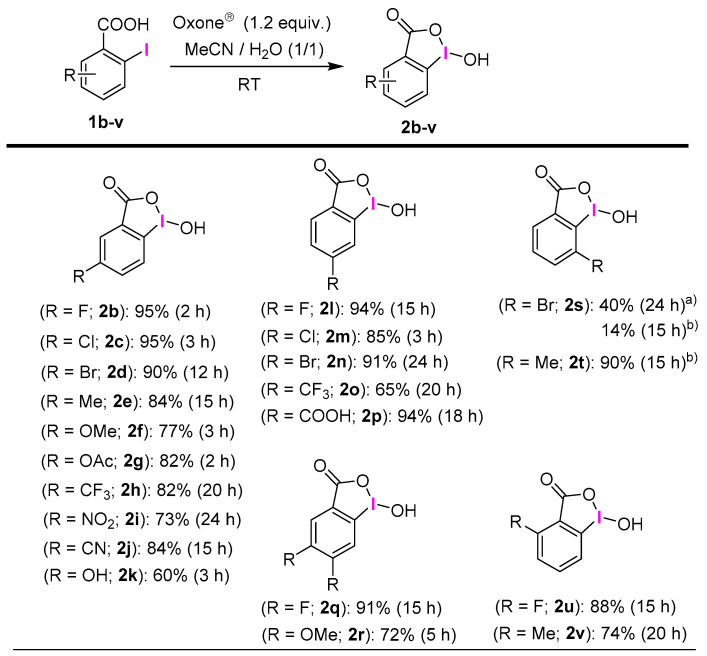
Substrate scope of the selective preparation of IBAs **2b**–**v** using Oxone^®^ under aqueous conditions. (a) This reaction was performed at 30 °C in the presence of 1.2 equivalents of Oxone^®^. (b) Performed using 5 equivalents of Oxone^®^ at 40 °C.

**Scheme 1:**
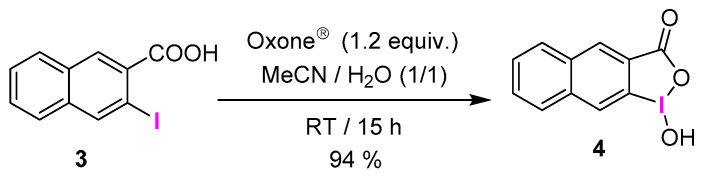
Synthesis of tricyclic hypervalent iodine compound **4**.

Next, we investigated the substrate scope for the synthesis of IBAs using Oxone^®^ under aqueous conditions with MeCN, and the results are shown in Figure 4. MeCN was used as a component of the solvent to dissolve substrates **1**. By oxidation of 5-substituted 2-IBs, IBAs **2b**–**d** containing fluoro-, chloro-, and bromo-substituents were smoothly obtained in excellent yields from the corresponding halo-substituted 2-IBs **1b**–**d**. From 2-IBs **1e**–**j** with electron-donating groups such as methyl-, methoxy-, and acethoxy-substituents (**1e**–**g**) and electron-withdrawing groups such as trifluoromethyl-, nitro- and cyano-substituents (**1h**–**j**), the desired IBAs **2e**–**j** were also produced in good yields. However, 2-IB bearing a hydroxy-substituent **1k** afforded the desired product **2k** in a moderate yield under the same conditions. In the oxidation of 4-substituted 2-IBs, fluoro-, chloro-, bromo-, trifluoromethyl-, and carboxy-substituted IBAs **2l**–**p** were obtained in excellent yields from the corresponding 2-IBs **1l**–**p**. In addition, the oxidized products of 4,5-disubstituted 2-IBs containing difluoro-substituents **2q** and dimethoxy-substituents **2r** were obtained in high yields. Meanwhile, with regard to 3-substituted 2-IBs, the reaction of methyl-substituted **1t** with a slight excess of Oxone^®^ afforded the expected IBA **2t** in a good yield, whereas the yield of bromo-substituted IBA **2s** was lower even at the elevated temperature and in the presence of a large excess of Oxone^®^. Steric effects are probably important in the formation of the cyclic λ^3^-iodanes. Indeed, the presence of a substituent at the *ortho* position of the iodine atom (3-position) interfered in the synthesis of the corresponding product for 3-bromo-substituted 2-IB **1s**. In the case of 6-substituted 2-IBs, fluoro-substituted IBA **2u** and methyl-substituted **2v** were obtained in good yields. Finally, the reaction of 3-iodonaphthalene-2-carboxylic acid **3** under the present conditions led to the expected tricyclic hypervalent iodine compound **4** in an excellent yield (Scheme 1).

### 2.2. IBAs Synthesis Using Ferric Effect

**Scheme 2:**
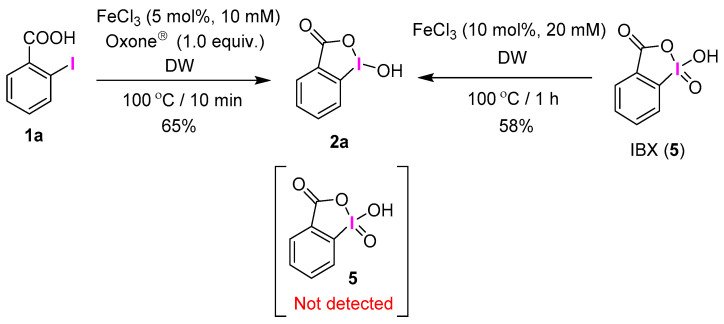
Effect of a ferric salt on the selective synthesis of IBA **2a**.

As mentioned in Section 2.1, our present method can selectively afford trivalent cyclic hypervalent iodine IBA at room temperature without contamination by pentavalent iodine byproduct. The mild conditions used contributed favorably to this product selectivity. Interestingly, we further found that iron ion in tap water (TW), which contained iron ion (5.8 μM or less), contribute to the IBA formation, whereas calcium and magnesium ions as main minerals in TW do not affect the selectivity. Indeed, IBA **2a** was selectively produced from 2-IB **1a** by heating even at 100 °C in DW containing 5 mol% FeCl_3_ (Scheme 2, left). On the other hand, IBX was instead formed as a main product in the absence of FeCl_3_ in deionized water (DW) [93]. Other ferric salts such as Fe(NO_3_), Fe(OTf)_3_, and FeSO_4_ had similar effects. In addition, it was found that pentavalent IBX **5** was converted to IBA **2a** in the presence of a catalytic amount of FeCl_3_ at 100 °C (Scheme 2, right), while the formation of unidentified high- and low-polar decomposition products were detected in the water and the acetone washing solution, respectively. Here, 2-IB **1a** was not produced. These results would indicate that overoxidation of IBA **2a** to pentavalent IBX **5** was strongly prevented by ferric salts. Thus, the effect of the metal ion in the decomposition of hazardous IBX **5** is also a significant key factor to ensure the safety for our trivalent cyclic hypervalent iodine synthesis under heating conditions.

**Figure 5:**
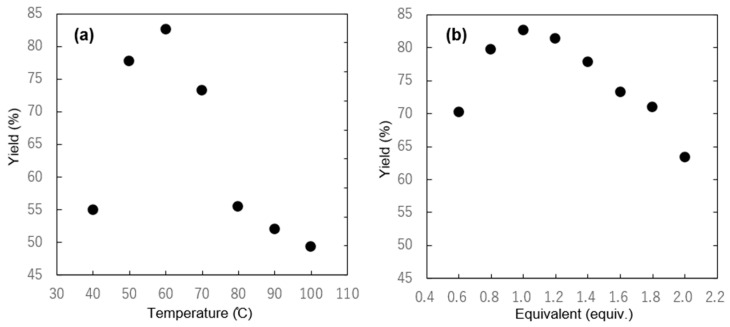
Optimization of (**a**) temperature using 1.0 equivalent of Oxone^®^ and (**b**) amount of Oxone^®^ at 60 °C for the synthesis of IBA **2a** from 2-IB **1a** in the presence of 2.5 mol% FeCl_3_ (MeCN/DW (1/1)).

The reaction time in the synthesis of IBAs was significantly shortened by heating. In the investigation of the heating conditions for the synthesis of IBA **2a** in 0.2 M 2-IB **1a** in the presence of 2.5 mol% FeCl_3_ for 10 min, the required amount of Oxone^®^ and the reaction temperature were thus optimized to 60 °C (Figure 5a) and 1.0 equivalent (Figure 5b), respectively. The yield of IBA **2a** was very sensitive to the reaction time, which dropped from 83% for 10 min to 70% for 1 h. IBA may be decomposed to small molecules in the presence of excess Oxone^®^; it has been reported that Oxone^®^ causes oxidative cleavage of the aromatic ring [95]. Without Oxone^®^, we also confirmed that IBA **2a** was hardly decomposed under the conditions of Scheme 2 in the presence of 1.0 equivalent of H_2_SO_4_ and 10 mol% FeCl_3_ at 100 °C for 10 min, while only 64% of IBA **2a** was recovered by replacing H_2_SO_4_ in the presence of Oxone^®^ under the same conditions. Thus, the excess uses of Oxone^®^ and performing the reaction at high temperature would decrease the IBA yield as shown in Figure 5a,b.

**Scheme 3:**
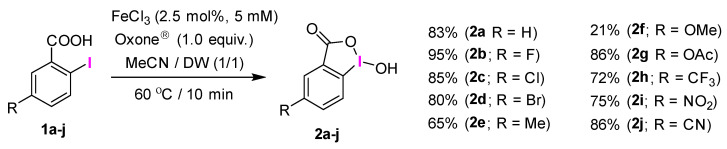
Short-time selective synthesis of IBAs **2a–j** in the presence of ferric salt.

This optimized heating method could be applied to the synthesis of IBAs **2a**–**j** (Scheme 3). 2-IB **1a** as well as the substrates **1b**–**d** and **1g**–**j** that are tolerable to over-oxidation at this temperature were successfully converted to the desired IBAs **2a**–**d** and **2g**–**j** in high yields. However, the transformation of 2-IB having an electron-rich functional group, i.e., methoxy-substituted 2-IB **1f**, resulted in low yield of the corresponding IBA **2f** due to the formation of 2-carboxy-*p*-benzoquinone by the oxidation with Oxone^®^. Therefore, in order to apply the heating conditions, the stability of the product to oxidation must be considered.

### 2.3. IBA Derivatives

**Figure 6:**
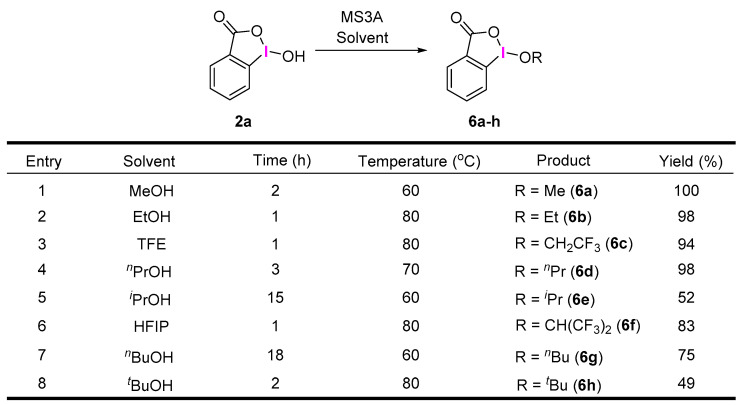
Benziodoxole alkoxides **6a**–**h** by solvolytic functionalization of IBA **2a**.

As previously mentioned, when IBA **2a** was synthesized in an aqueous solution with alcohols, alkoxy-substituted derivatives **3a**–**c** were obtained as byproducts by substitution of the hydroxyl ligand of IBA **2a** (see Figure 3), implying the potential of the solvolytic ligand exchange of IBA **2a** under mild conditions. For the ligand derivatization of IBAs, the water molecule is an obstacle because the ligand exchanges of the IBA hydroxy group are reversible. Thus, molecular sieves with a pore diameter of 3 Å (MS3Å) was used for the solvolytic functionalization of IBA **2a** in dehydrated protic solvent (Figure 6). The quantitative derivatization to benziodoxole methoxide (IB-OMe) **6a** was achieved by heating IBA **2a** at 60 °C in MeOH (Figure 6, entry 1). Upon treatment at 80 °C, benziodoxole ethoxide (IB-OEt) **6b** and benziodoxole 2,2,2-trifluoroethoxide (IB-OCH_2_CF_3_) **6c** were also produced in high yields by the ligand exchange reaction with EtOH and TFE, respectively (Figure 6, entries 2 and 3). Benziodoxole *n*-propoxide (IB-O^n^Pr) **6d** was obtained in 98% yield using *^n^*PrOH at 70 °C (Figure 6, entry 4), and benziodoxole isopropoxide (IB-O^i^Pr) **6e** was produced in 52% yield at 60 °C in the presence of *^i^*PrOH (Figure 6, entry 5). In the cases of IB-O^n^Pr **6d** and IB-O^i^Pr **6e**, the temperature control was essential to suppress the formation of a 2-IB-IBA condensate as a byproduct; here, the formation of 2-IB **1a** can be explained in terms of the alcohol oxidation by IBA. It is known that secondary alcohols are readily oxidized by IBA [83]. No unwanted byproduct was found during the transformation to benziodoxole hexafluoroisopropoxide (IB-OCH(CF_3_)_2_) **6f** using hexafluoroisopropanol (HFIP) at 80 °C (Figure 6, entry 6), which is most likely due to the stability of HFIP against oxidation. Indeed, the condensate between 2-IB and IBA appeared during the reaction for benziodoxole *n*-buthoxide (IB-O^n^Bu) **6g** using an oxidizable primary alcohol, *^n^*BuOH, at 80 °C, whereas such byproduct was not observed in the synthesis of benziodoxole *tert*-buthoxide (IB-O^t^Bu) **6h** using *^t^*BuOH as a solvent inert to oxidation. Nevertheless, IB-O^n^Bu **6g** could be selectively obtained by heat treatment at 60 °C without the formation of the condensate.

**Scheme 4:**
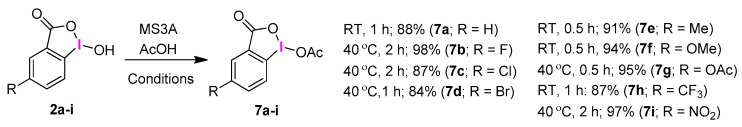
Transformation of IBAs **2a**–**i** to benziodoxole acetates **7a**–**i**.

Using AcOH as a solvent, the solvolytic method was further applied to the synthesis of benziodoxole acetate (IB-OAc) **7a** and its analogs (R-IB-OAc) **7b**–**i** from the corresponding IBAs **2a**–**i** (Scheme 4). IB-OAc **7a** was easily produced in good yield by ligand exchange of IBA **2a** with AcOH at room temperature. Similarly, these transformations successfully afforded R-IB-OAc **7b**–**d** containing fluoro-, chloro-, and bromo-substituents; **7e**–**g** with electron-donating methyl-, methoxy-, and acethoxy-groups; and **7h** and **7i** bearing an electron-withdrawing trifluoromethyl- and nitro-substituent, respectively.

## 3. Materials and Methods

### 3.1. General Information

Substrates **1i** [96], **1k** [97], **1n** [98], **1o** [98], **1p** [99], **1q** [100], **1s** [101], **1t** [98], **1v** [102], and **3** [65] were prepared by Sandmeyer reaction of the corresponding anthranilic acids. Substrate **1g** [84] was synthesized by acetylation of compound **1k**. Substrate **1j** [103] is derived from 5-bromo-anthranilic acid methyl ester. ^1^H, ^13^C, and ^19^F nuclear magnetic resonance (NMR) spectra were recorded on ECS 400 and ECX 500 NMR spectrometers (JEOL Ltd., Tokyo, Japan) using deuterated dimethyl sulfoxide (DMSO-d6) or chloroform (CDCl_3_) as a solvent. Chemical shifts (δ) are reported in parts per million (ppm) relative to tetramethylsilane (δ = 0 ppm) as an internal standard for ^1^H and ^13^C NMR spectra and hexafluoroacetone (δ = −84.6 ppm) as an internal standard for ^19^F NMR spectra. Coupling constants (*J*) are reported in Hertz (Hz), and the multiplicity is reported according to the following convention: singlet (s), doublet (d), double doublet (dd), double double doublet (ddd), double triplet (dt), triplet (t), triple doublet (td), quartet (q), quintet (quin), sextet (sext), septet (sep), and multiplet (m). Data are reported as follows: Chemical shift (number of protons, multiplicity, coupling constants). Infrared (IR) spectra were recorded on a JASCO FT/IR-4200 spectrometer (JASCO Co., Tokyo, Japan) on diffuse reflectance method using KBr powder. Absorptions are expressed in reciprocal centimeter (cm^−1^). High resolution mass spectra (HRMS) obtained by the direct analysis in real time (DART) method were recorded on a Thermo Scientific Exactive Plus Orbitrap (Thermo Fisher Scientific, Inc., Waltham, MA, USA).

### 3.2. Synthesis of IBA Analogues

#### 3.2.1. General Procedure for the Synthesis of IBAs **2a**–**v** and **4**

To a solution of 2-IBs (1.0 mmol) in MeCN (5 mL) was added Oxone^®^ (738 mg, 1.2 mmol) and H_2_O (TW for Figure 3; Figure 4, Scheme 1 or DW for Scheme 2; Scheme 3, 5 mL). After the mixture was stirred at room temperature for the appropriate time (see Figure 4 and Scheme 1), the product was filtered under reduced pressure. The residue was washed with water and acetone to obtain the corresponding IBAs **2a**–**v** and **4** (see Supplementary Materials for ^1^H NMR spectroscopic data) as a white powder.

#### 3.2.2. 1-Hydroxy-1λ^3^-benzo[d][1,2]iodaoxol-3(1H)-one (**2a**)

^1^H NMR (400 MHz, DMSO-*d6*): δ 7.72 (1H, td, *J* = 7.3, 0.9 Hz, H5), 7.86 (1H, d, *J* = 8.2 Hz, H3), 7.97 (1H, ddd, *J* = 8.7, 7.4, 1.8 Hz, H4), 8.03 (1H, dd, *J* = 7.8, 1.4 Hz, H6), 8.08 (1H, s) ppm. ^13^C NMR (100 MHz, DMSO-*d6*): δ 120.3 (C2), 126.2 (C3), 130.3 (C5), 131.0 (C1), 131.4 (C6), 134.4 (C4), 167.7 (COOH) ppm. IR (ATR, KBr): *ν* 2936 (OH), 1616 (C=O), 1566 (C=O) cm^−1^. Mp: 243–244 °C. ^1^H and ^13^C NMR data are consistent with those reported in the literature [33].

#### 3.2.3. 5-Fluoro-1-hydroxy-1λ^3^-benzo[d][1,2]iodaoxol-3(1H)-one (**2b**)

^1^H NMR (500 MHz, DMSO-*d6*): δ 7.76 (1H, dd, *J* = 8.3, 2.6 Hz), 7.79–7.88 (2H, m), 8.21 (1H, s) ppm. ^13^C NMR (125 MHz, DMSO-*d6*): δ 114.2, 117.3 (d, *J* = 22.7 Hz), 121.7 (d, *J* = 23.8 Hz), 128.3 (d, *J* = 8.4 Hz), 134.1 (d, *J* = 7.2 Hz), 163.9 (d, *J* = 246.8 Hz), 166.4 (d, *J* = 2.4 Hz) ppm. ^19^F NMR (470 MHz, DMSO-*d6*): δ −116.2 (dt, *J* = 5.7, 8.6 Hz) ppm. IR (ATR, KBr): *ν* 2904 (OH), 1635 (C=O), 1577 (C=O) cm^−1^. Mp: 241–242 °C. ^1^H and ^13^C NMR data are consistent with those reported in the literature [104].

#### 3.2.4. 5-Chloro-1-hydroxy-1λ^3^-benzo[d][1,2]iodaoxol-3(1H)-one (**2c**)

^1^H NMR (400 MHz, DMSO-*d6*): δ 7.81 (1H, d, *J* = 8.7 Hz), 7.95 (1H, d, *J* = 2.3 Hz), 8.03 (1H, dd, *J* = 8.7, 2.3 Hz), 8.28 (1H, s) ppm. ^13^C NMR (100 MHz, DMSO-*d6*): δ 118.6, 128.1, 130.3, 133.5, 134.0, 135.8, 166.3 ppm. IR (ATR, KBr): *ν* 2905 (OH), 1624 (C=O), 1560 (C=O) cm^−1^. Mp: 294–295 °C. ^1^H and ^13^C NMR data are consistent with those reported in the literature [105].

#### 3.2.5. 5-Bromo-1-hydroxy-1λ^3^-benzo[d][1,2]iodaoxol-3(1H)-one (**2d**)

^1^H NMR (400 MHz, DMSO-*d6*): δ 7.74 (1H, d, *J* = 8.7 Hz), 8.07 (1H, d, *J* = 2.3 Hz), 8.15 (1H, dd, *J* = 8.7, 2.3 Hz), 8.27 (1H, s) ppm. ^13^C NMR (100 MHz, DMSO-*d6*): δ 119.5, 124.2, 128.3, 133.3, 133.7, 136.8, 166.2 ppm. IR (ATR, KBr): *ν* 2884 (OH), 1617 (C=O), 1557 (C=O) cm^−1^. Mp: 236–238 °C. ^1^H and ^13^C NMR data are consistent with those reported in the literature [106].

#### 3.2.6. 1-Hydroxy-5-methyl-1λ^3^-benzo[d][1,2]iodaoxol-3(1H)-one (**2e**)

^1^H NMR (400 MHz, DMSO-*d6*): δ 2.48 (3H, s), 7.70 (1H, d, *J* = 8.2 Hz), 7.79 (1H, dd, *J* = 8.7, 1.8 Hz), 7.85 (1H, s), 8.01 (1H, s) ppm. ^13^C NMR (100 MHz, DMSO-*d6*): δ 20.1, 116.7, 125.9, 131.3, 131.4, 135.2, 140.4, 167.7 ppm. IR (ATR, KBr): *ν* 3054 (OH), 1622 (C=O), 1569 (C=O) cm^−1^. Mp: 212–214 °C. ^1^H and ^13^C NMR data are consistent with those reported in the literature [105].

#### 3.2.7. 1-Hydroxy-5-methoxy-1λ^3^-benzo[d][1,2]iodaoxol-3(1H)-one (**2f**)

^1^H NMR (400 MHz, DMSO-*d6*): δ 3.89 (3H, s), 7.52 (1H, d, *J* = 2.7 Hz), 7.55 (1H, dd, *J* = 8.7, 2.8 Hz), 7.67 (1H, d, *J* = 9.2 Hz), 8.04 (1H, s) ppm. ^13^C NMR (100 MHz, DMSO-*d6*): δ 55.8, 108.9, 114.8, 121.5, 127.0, 132.9, 161.4, 167.4 ppm. IR (ATR, KBr): *ν* 2953 (OH), 1620 (C=O), 1577 (C=O) cm^−1^. Mp: 217–218 °C. ^1^H and ^13^C NMR data are consistent with those reported in the literature [105].

#### 3.2.8. 1-Hydroxy-3-oxo-1,3-dihydro-1λ^3^-benzo[d][1,2]iodaoxol-5-yl acetate (**2g**)

^1^H NMR (500 MHz, DMSO-*d6*): δ 2.33 (3H, s), 7.74 (1H, dd, *J* = 8.6, 2.3 Hz), 7.77 (1H, d, *J* = 2.3 Hz), 7.84 (1H, d, *J* = 8.6 Hz), 8.16 (1H, s) ppm. ^13^C NMR (125 MHz, DMSO-*d6*): δ 20.8, 116.1, 124.2, 127.4, 127.9, 133.0, 152.5, 166.8, 169.0 ppm. IR (ATR, KBr): *ν* 2891 (OH), 1759 (C=O), 1604 (C=O), 1559 (C=O) cm^−1^. Mp: 207–208 °C. HRMS (DART, m/z) calcd for C_9_H_8_IO_5_ [M + H]^+^: 322.9411; found: 322.9413.

#### 3.2.9. 1-Hydroxy-5-(trifluoromethyl)-1λ^3^-benzo[d][1,2]iodaoxol-3(1H)-one (**2h**)

^1^H NMR (500 MHz, DMSO-*d6*): δ 8.08 (1H, d, *J* = 8.0 Hz), 8.21 (1H, s), 8.33 (1H, d, *J* = 8.1 Hz), 8.38 (1H, s) ppm. ^13^C NMR (125 MHz, DMSO-*d6*): δ 123.4 (q, *J* = 271.0 Hz), 125.5, 127.1 (d, *J* = 3.6 Hz), 127.9, 130.6 (d, *J* = 2.4 Hz), 131.6 (q, *J* = 32.6 Hz), 132.9, 166.3 ppm. ^19^F NMR (470 MHz, DMSO-*d6*): δ −64.5 ppm. IR (ATR, KBr): *ν* 2854 (OH), 1597 (C=O), 1559 (C=O) cm^−1^. Mp: 233–235 °C. HRMS (DART, m/z) calcd for C_8_H_5_F_3_IO_3_ [M + H]^+^: 332.9230; found: 332.9227.

#### 3.2.10. 1-Hydroxy-5-nitro-1λ^3^-benzo[d][1,2]iodaoxol-3(1H)-one (**2i**)

^1^H NMR (400 MHz, DMSO-*d6*): δ 8.10 (1H, d, *J* = 8.7 Hz), 8.54 (1H, s), 8.57 (1H, d, *J* = 2.3 Hz), 8.73 (1H, dd, *J* = 8.7, 2.8 Hz) ppm. ^13^C NMR (100 MHz, DMSO-*d6*): δ 124.8, 127.7, 128.1, 128.2, 133.4, 149.7, 165.9 ppm. IR (ATR, KBr): *ν* 2834 (OH), 1617 (C=O), 1572 (C=O), 1541 (C=O) cm^−1^. Mp: 214–216 °C. ^1^H and ^13^C NMR data are consistent with those reported in the literature [107].

#### 3.2.11. 1-Hydroxy-3-oxo-1,3-dihydro-1λ^3^-benzo[d][1,2]iodaoxole-5-carbonitrile (**2j**)

^1^H NMR (500 MHz, DMSO-*d6*): δ 8.01 (1H, d, *J* = 8.6 Hz), 8.32–8.41 (3H, m) ppm. ^13^C NMR (125 MHz, DMSO-*d6*): δ 113.5, 117.3, 126.4, 127.7, 132.9, 134.2, 137.0, 166.0 ppm. IR (ATR, KBr): *ν* 2903 (OH), 1625 (C=O), 1582 (C=O), 1561 (C=O) cm^−1^. Mp: 234–236 °C. HRMS (DART, m/z) calcd for C_8_H_5_INO_3_ [M + H]^+^: 289.9309; found: 289.9310.

#### 3.2.12. 1,5-Dihydroxy-1λ^3^-benzo[d][1,2]iodaoxol-3(1H)-one (**2k**)

^1^H NMR (400 MHz, DMSO-*d6*): δ 7.36 (1H, dd, *J* = 8.7, 2.7 Hz), 7.40 (1H, d, *J* = 2.3 Hz), 7.57 (1H, d, *J* = 9.2 Hz), 7.94 (1H, s) ppm. ^13^C NMR (100 MHz, DMSO-*d6*): δ 106.6, 117.1, 122.0, 127.0, 132.8, 159.7, 167.6 ppm. IR (ATR, KBr): *ν* 3447 (OH), 3234 (OH), 1576 (C=O) cm^−1^. Mp: 230–232 °C. HRMS (DART, m/z) calcd for C_7_H_6_IO_4_ [M + H]^+^: 280.9305; found: 280.9304.

#### 3.2.13. 6-Fluoro-1-hydroxy-1λ^3^-benzo[d][1,2]iodaoxol-3(1H)-one (**2l**)

^1^H NMR (500 MHz, DMSO-*d6*): δ 7.53–7.60 (2H, m), 8.01 (1H, dd, *J* = 8.0, 5.2 Hz), 8.21 (1H, s) ppm. ^13^C NMR (125 MHz, DMSO-*d6*): δ 113.5 (d, *J* = 27.4 Hz), 118.0 (d, *J* = 22.7 Hz), 122.8 (d, *J* = 8.4 Hz), 128.3, 132.9 (d, *J* = 8.4 Hz), 166.0 (d, *J* = 254.0 Hz), 166.7 ppm. ^19^F NMR (470 MHz, DMSO-*d6*): δ −109.0 (dt, *J* = 5.8, 8.6 Hz) ppm. IR (ATR, KBr): *ν* 3091 (OH), 1636 (C=O), 1586 (C=O) cm^−1^. Mp: 206–208 °C. ^1^H and ^13^C NMR data are consistent with those reported in the literature [105].

#### 3.2.14. 6-Chloro-1-hydroxy-1λ^3^-benzo[d][1,2]iodaoxol-3(1H)-one (**2m**)

^1^H NMR (400 MHz, DMSO-*d6*): δ 7.75 (1H, d, *J* = 1.8 Hz), 7.78 (1H, dd, *J* = 7.8, 1.8 Hz), 7.96 (1H, d, *J* = 8.2, 2.3 Hz), 8.27 (1H, s) ppm. ^13^C NMR (100 MHz, DMSO-*d6*): δ 122.1, 125.7, 130.6, 130.7, 132.2, 139.3, 166.7 ppm. IR (ATR, KBr): *ν* 2854 (OH), 1607 (C=O), 1557 (C=O) cm^−1^. Mp: 212–214 °C. ^1^H NMR data is consistent with those reported in the literature [94].

#### 3.2.15. 6-Bromo-1-hydroxy-1λ^3^-benzo[d][1,2]iodaoxol-3(1H)-one (**2n**)

^1^H NMR (500 MHz, DMSO-*d6*): δ 7.80–7.98 (3H, m), 8.23 (1H, s) ppm. ^13^C NMR (125 MHz, DMSO-*d6*): δ 122.1, 127.9, 128.5, 131.0, 132.5, 133.5, 166.8 ppm. IR (ATR, KBr): *ν* 2844 (OH), 1602 (C=O), 1556 (C=O) cm^−1^. Mp: 222–224 °C. HRMS (DART, m/z) calcd for C_7_H_5_BrIO_3_ [M + H]^+^: 342.8461; found: 342.8460.

#### 3.2.16. 1-Hydroxy-6-(trifluoromethyl)-1λ^3^-benzo[d][1,2]iodaoxol-3(1H)-one (**2o**)

^1^H NMR (400 MHz, DMSO-*d6*): δ 8.06 (1H, s), 8.10 (1H, d, *J* = 8.1 Hz), 8.20 (1H, d, *J* = 8.0 Hz), 8.38 (1H, s) ppm. ^13^C NMR (100 MHz, DMSO-*d6*): δ 121.7, 123.1 (d, *J* = 3.6 Hz), 123.4 (d, *J* = 270.7 Hz), 127.6 (d, *J* = 3.6 Hz), 131.9, 133.9 (q, *J* = 32.2 Hz), 135.4, 166.4 ppm. ^19^F NMR (370 MHz, DMSO-*d6*): δ −64.6 ppm. IR (ATR, KBr): *ν* 2871 (OH), 1616 (C=O), 1560 (C=O) cm^−1^. Mp: 216–217 °C. ^1^H and ^13^C NMR data are consistent with those reported in the literature [12].

#### 3.2.17. 1-Hydroxy-3-oxo-1,3-dihydro-1λ^3^-benzo[d][1,2]iodaoxole-6-carboxylic acid (**2p**)

^1^H NMR (500 MHz, DMSO-*d6*): δ ppm. ^13^C NMR (125 MHz, DMSO-*d6*): δ ppm. IR (ATR, KBr): *ν* 2832 (OH), 1704 (C=O), 1616 (C=O), 1558 (C=O) cm^−1^. Mp: 291–293 °C. HRMS (DART, m/z) calcd for C_8_H_6_IO_5_ [M + H]^+^: 308.9254; found: 308.9252.

#### 3.2.18. 5,6-Difluoro-1-hydroxy-1λ^3^-benzo[d][1,2]iodaoxol-3(1H)-one (**2q**)

^1^H NMR (500 MHz, DMSO-*d6*): δ 7.73 (1H, dd, *J* = 9.2, 6.9 Hz), 7.97 (1H, dd, *J* = 9.7, 7.4 Hz), 8.37 (1H, s) ppm. ^13^C NMR (125 MHz, DMSO-*d6*): δ 115.5 (d, *J* = 22.7 Hz), 119.2 (d, *J* = 19.1 Hz), 129.16 (d, *J* = 2.4 Hz), 129.20 (d, *J* = 3.6 Hz), 151.3 (dd, *J* = 263.5, 13.1 Hz), 153.7 (dd, *J* = 256.4, 14.3 Hz), 165.9 ppm. ^19^F NMR (470 MHz, DMSO-*d6*): δ −138.7–138.5 (m), −132.6–132.4 (m) ppm. IR (ATR, KBr): *ν* 2895 (OH), 1624 (C=O), 1591 (C=O) cm^−1^. Mp: 201–203 °C. HRMS (DART, m/z) calcd for C_7_H_4_F_2_IO_3_ [M + H]^+^: 300.9168; found: 300.9170.

#### 3.2.19. 1-Hydroxy-5,6-dimethoxy-1λ^3^-benzo[d][1,2]iodaoxol-3(1H)-one (**2r**)

^1^H NMR (500 MHz, DMSO-*d6*): δ 3.89 (6H, s), 7.24 (1H, s), 7.46 (1H, s), 7.95 ppm. ^13^C NMR (125 MHz, DMSO-*d6*): δ 55.9, 56.0, 107.4, 110.7, 112.4, 123.9, 150.6, 154.1, 167.8 ppm. IR (ATR, KBr): *ν* 3016 (OH), 1592 (C=O), 1559 (C=O) cm^−1^. Mp: 201–203 °C. ^1^H and ^13^C NMR data are consistent with those reported in the literature [46].

#### 3.2.20. 7-Bromo-1-hydroxy-1λ^3^-benzo[d][1,2]iodaoxol-3(1H)-one (**2s**)

^1^H NMR (500 MHz, DMSO-*d6*): δ 7.60 (1H, t, *J* = 7.7 Hz), 7.97 (1H, dd, *J* = 7.7, 1.5 Hz), 8.02 (1H, dd, *J* = 7.5, 1.2 Hz) ppm. ^13^C NMR (125 MHz, DMSO-*d6*): δ 119.6, 130.0, 133.1, 135.4, 140.5, 146.1, 167.0 ppm. IR (ATR, KBr): *ν* 3273 (OH), 1647 (C=O) cm^−1^. Mp: 154–155 °C. HRMS (DART, m/z) calcd for C_7_H_5_BrIO_3_ [M + H]^+^: 342.8461; found: 342.8462.

#### 3.2.21. 1-Hydroxy-7-methyl-1λ^3^-benzo[d][1,2]iodaoxol-3(1H)-one (**2t**)

^1^H NMR (400 MHz, DMSO-*d6*): δ 2.79 (3H, s), 7.57–7.73 (2H, m), 7.90 (1H, d, *J* = 6.9 Hz) ppm. ^13^C NMR (100 MHz, DMSO-*d6*): δ 19.6, 128.7, 132.0, 132.6, 137.9, 139.1, 147.4, 167.9 ppm. IR (ATR, KBr): *ν* 1672 (C=O) cm^−1^. Mp: 164–166 °C. ^1^H NMR data are consistent with those reported in the literature [106].

#### 3.2.22. 4-Fluoro-1-hydroxy-1λ^3^-benzo[d][1,2]iodaoxol-3(1H)-one (**2u**)

^1^H NMR (400 MHz, DMSO-*d6*): δ 7.51 (1H, dd, *J* = 10.1, 8.2 Hz), 7.71 (1H, d, *J* = 7.7 Hz), 7.90 (1H, td, *J* = 8.2, 4.6 Hz), 8.24 (1H, s) ppm. ^13^C NMR (100 MHz, DMSO-*d6*): δ 118.6 (d, *J* = 22.0 Hz), 119.2 (d, *J* = 11.5 Hz), 122.5 (d, *J* = 3.8 Hz), 123.2, 134.3 (d, *J* = 8.6 Hz), 163.8 (d, *J* = 4.8 Hz), 163.8 (d, *J* = 264.4 Hz) ppm. ^19^F NMR (375 MHz, DMSO-*d6*): δ −114.7 (dd, *J* = 15.2, 4.9 Hz) ppm. IR (ATR, KBr): *ν* 3091 (OH), 1636 (C=O), 1586 (C=O) cm^−1^. Mp: 213–214 °C. ^1^H and ^13^C NMR data are consistent with those reported in the literature [106,108].

#### 3.2.23. 1-Hydroxy-4-methyl-1λ^3^-benzo[d][1,2]iodaoxol-3(1H)-one (**2v**)

^1^H NMR (400 MHz, DMSO-*d6*): δ 2.70 (3H, s), 7.48–7.55 (1H, m), 7.72–7.80 (2H, m), 7.94 (1H, s) ppm. ^13^C NMR (100 MHz, DMSO-*d6*): δ 20.4, 122.2, 124.2, 128.2, 133.3, 133.4, 144.3, 168.0 ppm. IR (ATR, KBr): *ν* 2926 (OH), 1625 (C=O), 1584 (C=O) cm^−1^. Mp: 212–213 °C. ^1^H and ^13^C NMR data are consistent with those reported in the literature [106].

#### 3.2.24. 1-Hydroxy-1λ^3^-naphtho[2,3-d][1,2]iodaoxol-3(1H)-one (**4**)

^1^H NMR (400 MHz, DMSO-*d6*): δ 7.76 (2H, m), 8.14–8.33 (2H, m), 8.29 (1H, d, *J* = 8.2 Hz), 8.39 (1H, s), 8.69 (1H, s) ppm. ^13^C NMR (100 MHz, DMSO-*d6*): δ 115.9, 126.3, 127.8, 127.9, 128.1, 128.9, 129.3, 131.7, 132.8, 135.8, 167.7 ppm. IR (ATR, KBr): *ν* 3053 (OH), 1698 (C=O), 1607 (C=O), 1559 (C=O) cm^−1^. Mp: 164–165 °C. ^1^H and ^13^C NMR data are consistent with those reported in the literature [107].

### 3.3. Synthesis of Benziodoxole Alkoxides

#### 3.3.1. General Procedure for the Synthesis of Benziodoxole Alkoxides (**6**)

To a suspension of IBA **2a** (264 mg, 1.0 mmol) in an appropriate alcohol (10 mL) was added MS3Å (1 g). After the mixture was stirred under the appropriate conditions (see Figure 6), MS3Å was filtered using CH_2_Cl_2_, and the solvents were then removed by evaporation. The residue was washed with hexane and filtered to remove the corresponding alcohol completely. The residue was dissolved with CH_2_Cl_2_ and the extract was then filtered through filter paper to remove unreacted substrate. Removal of the solvent by evaporation gave the corresponding benziodoxole alkoxides **6a**–**h** as a white powder.

#### 3.3.2. 1-Methoxy-1λ^3^-benzo[d][1,2]iodaoxol-3(1H)-one (**6a**)

^1^H NMR (500 MHz, CDCl_3_): δ 4.29 (3H, s), 7.70 (1H, t, *J* = 7.7 Hz), 7.78 (1H, d, *J* = 8.1 Hz), 7.91 (1H, ddd, *J* = 8.6, 6.9, 1.2 Hz), 8.28 (1H, dd, *J* = 7.5, 1.2 Hz) ppm. ^13^C NMR (125 MHz, CDCl_3_): δ 62.3, 118.6, 126.0, 130.6, 131.0, 132.9, 135.1, 168.0 ppm. IR (ATR, KBr): *ν* 1653 (C=O) cm^−1^. Mp: 161–163 °C. ^1^H and ^13^C NMR data are consistent with those reported in the literature [28,109].

#### 3.3.3. 1-Ethoxy-1λ^3^-benzo[d][1,2]iodaoxol-3(1H)-one (**6b**)

^1^H NMR (500 MHz, CDCl_3_): δ 1.35 (3H, t, *J* = 6.9 Hz), 4.30 (2H, q, *J* = 6.9 Hz), 7.70 (1H, t, *J* = 7.4 Hz), 7.79 (1H, d, *J* = 8.9 Hz), 7.89 (1H, td, *J* = 8.6, 1.8 Hz), 8.28 (1H, dd, *J* = 7.2, 2.0 Hz) ppm. ^13^C NMR (125 MHz, CDCl_3_): δ 19.0, 69.9, 118.8, 125.9, 130.7, 131.0, 132.9, 135.0, 168.0 ppm. IR (ATR, KBr): *ν* 1655 (C=O) cm^−1^. Mp: 123–125 °C. ^1^H and ^13^C NMR data are consistent with those reported in the literature [109].

#### 3.3.4. 1-(2,2,2-trifluoroethoxy)-1λ^3^-benzo[d][1,2]iodaoxol-3(1H)-one (**6c**)

^1^H NMR (500 MHz, CDCl_3_): δ 4.52 (2H, q, *J* = 8.6 Hz), 7.74 (1H, t, *J* = 7.5 Hz), 7.86 (1H, d, *J* = 8.0 Hz), 7.97 (1H, ddd, *J* = 8.0, 6.9, 1.2 Hz), 8.27 (1H, dd, *J* = 7.5, 1.2 Hz) ppm. ^13^C NMR (125 MHz, CDCl_3_): δ 69.7 (q, *J* = 34.2 Hz), 119.0, 123.5 (q, *J* = 278.2 Hz), 126.5, 129.5, 131.4, 133.2, 135.9, 167.9 ppm. ^19^F NMR (470 MHz, CDCl_3_): δ −77.2 (q, *J* = 9.1 Hz) ppm. IR (ATR, KBr): *ν* 1646 (C=O) cm^−1^. Mp: 139–141 °C. HRMS (DART, m/z) calcd for C_9_H_7_F_3_IO_3_ [M + H]^+^: 346.9386; found: 346.9384.

#### 3.3.5. 1-Propoxy-1λ^3^-benzo[d][1,2]iodaoxol-3(1H)-one (**6d**)

^1^H NMR (500 MHz, CDCl_3_): δ 1.02 (3H, t, *J* = 7.5 Hz), 1.72 (2H, sext, *J* = 7.1 Hz), 4.20 (2H, t, *J* = 6.6 Hz), 7.70 (1H, t, *J* = 7.2 Hz), 7.79 (1H, d, *J* = 8.6 Hz), 7.89 (1H, t, *J* = 7.2 Hz), 8.28 (1H, d, *J* = 7.5 Hz) ppm. ^13^C NMR (125 MHz, CDCl_3_): δ 10.1, 26.5, 76.0, 118.9, 125.9, 130.7, 130.9, 132.8, 135.0, 167.9 ppm. IR (ATR, KBr): *ν* 1651 (C=O) cm^−1^. Mp: 146–148 °C. HRMS (DART, m/z) calcd for C_10_H_12_IO_3_ [M + H]^+^: 306.9826; found: 306.9823.

#### 3.3.6. 1-Isopropoxy-1λ^3^-benzo[d][1,2]iodaoxol-3(1H)-one (**6e**)

^1^H NMR (500 MHz, CDCl_3_): δ 1.36 (6H, d, *J* = 6.3 Hz), 4.33 (1H, sep, *J* = 6.1 Hz), 7.69 (1H, t, *J* = 7.4 Hz), 7.82 (1H, d, *J* = 7.5 Hz), 7.88 (1H, td, *J* = 7.8, 1.5 Hz), 8.28 (1H, dd, *J* = 7.5, 1.7 Hz) ppm. ^13^C NMR (125 MHz, CDCl_3_): δ 25.2, 75.6, 119.1, 126.0, 130.8, 130.9, 132.7, 134.8, 168.0 ppm. IR (ATR, KBr): *ν* 1653 (C=O) cm^−1^. Mp: 253–254 °C. ^1^H and ^13^C NMR data are consistent with those reported in the literature [109].

#### 3.3.7. 1-((1,1,1,3,3,3-hexafluoropropan-2-yl)oxy)-1λ^3^-benzo[d][1,2]iodaoxol-3(1H)-one (**6f**)

^1^H NMR (500 MHz, CDCl_3_): δ 4.80 (1H, sep, *J* = 5.8 Hz), 7.74 (1H, ddd, *J* = 8.0, 7.5, 1.4 Hz), 7.97–8.04 (2H, m), 8.24 (1H, dd, *J* = 7.4, 1.2 Hz) ppm. ^13^C NMR (125 MHz, CDCl_3_): δ 76.1 (quin, *J* = 32.5 Hz), 119.4, 122.2 (q, *J* = 283.8 Hz), 127.3, 128.5, 131.6, 133.3, 136.4, 168.1 ppm. ^19^F NMR (470 MHz, CDCl_3_): δ −76.0 (d, *J* = 5.7 Hz) ppm. IR (ATR, KBr): *ν* 1661 (C=O) cm^−1^. Mp: 148–149 °C. HRMS (DART, m/z) calcd for C_10_H_6_F_6_IO_3_ [M + H]^+^: 414.9260; found: 414.9258.

#### 3.3.8. 1-Butoxy-1λ^3^-benzo[d][1,2]iodaoxol-3(1H)-one (**6g**)

^1^H NMR (500 MHz, CDCl_3_): δ 0.98 (3H, t, *J* = 7.5 Hz), 1.46 (2H, sext, *J* = 7.4 Hz), 1.68 (2H, quin, *J* = 7.2 Hz), 4.24 (2H, t, *J* = 6.6 Hz), 7.70 (1H, t, *J* = 7.5 Hz), 7.78 (1H, d, *J* = 8.0 Hz), 7.89 (1H, t, *J* = 7.5 Hz), 8.28 (1H, d, *J* = 7.5 Hz) ppm. ^13^C NMR (125 MHz, CDCl_3_): δ 13.9, 19.0, 35.4, 74.2, 118.9, 125.9, 130.7, 131.0, 132.9, 135.0, 167.9 ppm. IR (ATR, KBr): *ν* 1650 (C=O) cm^−1^. Mp: 143–144 °C. ^1^H and ^13^C NMR data are consistent with those reported in the literature [110].

#### 3.3.9. 1-(tert-Butoxy)-1λ^3^-benzo[d][1,2]iodaoxol-3(1H)-one (**6h**)

^1^H NMR (500 MHz, CDCl_3_): δ 1.41 (9H, s), 7.67 (1H, ddd, *J* = 8.1, 7.5, 1.8 Hz), 7.83–7.91 (2H,m), 8.26 (1H, dd, *J* = 8.1, 1.8 Hz) ppm. ^13^C NMR (125 MHz, CDCl_3_): δ 30.4, 78.6, 119.6, 126.2, 130.8, 131.0, 132.4, 134.7, 168.0 ppm. IR (ATR, KBr): *ν* 1659 (C=O) cm^−1^. Decomp: 265 °C. HRMS (DART, m/z) calcd for C_11_H_13_IO_3_ [M + H]^+^: 320.9982; found: 320.9980.

### 3.4. Synthesis of Benziodoxole Acetates

#### 3.4.1. General Procedure for the Synthesis of Benziodoxole Acetates (**7**)

MS3Å (0.5 g) was added to a suspension of IBAs **2a**–**i** (0.50 mmol) in AcOH (5 mL), and the mixture was stirred under the appropriate conditions (see Scheme 4). Then, MS3Å was filtered using CH_2_Cl_2_, and the solvents were removed by evaporation. The residue was washed with ether and filtered to remove AcOH completely. The resulting residue was dissolved with CH_2_Cl_2_, and the extract was then filtered through filter paper to remove unreacted substrate. After solvent removal by evaporation, the corresponding benziodoxole acetates **7a**–**i** were obtained as a white powder.

#### 3.4.2. 3-Oxo-1λ^3^-benzo[d][1,2]iodaoxol-1(3H)-yl acetate (**7a**)

^1^H NMR (500 MHz, CDCl_3_): δ 2.27 (3H, s), 7.72 (1H, td, *J* = 7.5, 1.2 Hz), 7.94 (1H, ddd, *J* = 8.6, 6.9, 1.2 Hz), 8.01 (1H, d, *J* = 8.6 Hz), 8.25 (1H, dd, *J* = 7.5, 1.2 Hz) ppm. ^13^C NMR (125 MHz, CDCl_3_): δ 20.2, 118.3, 128.9, 129.2, 131.2, 133.1, 136.1, 168.1, 176.3 ppm. IR (ATR, KBr): *ν* 1684 (C=O) cm^−1^. Mp: 220–222 °C. ^1^H and ^13^C NMR data are consistent with those reported in the literature [109].

#### 3.4.3. 5-Fluoro-3-oxo-1λ^3^-benzo[d][1,2]iodaoxol-1(3H)-yl acetate (**7b**)

^1^H NMR (500 MHz, CDCl_3_): δ 2.26 (3H, s), 7.64 (1H, ddd, *J* = 8.6, 7.7, 2.9 Hz), 7.94–8.00 (2H, m) ppm. ^13^C NMR (125 MHz, CDCl_3_): δ 20.2, 111.3, 120.0 (d, *J* = 23.8 Hz), 123.7 (d, *J* = 22.7 Hz), 131.0 (d, *J* = 8.3 Hz), 131.8 (d, *J* = 7.2 Hz), 165.0 (d, *J* = 252.8 Hz), 166.8, 176.4 ppm. ^19^F NMR (470 MHz, CDCl_3_): δ −110.8 (td, *J* = 7.2, 4.3 Hz) ppm. IR (ATR, KBr): *ν* 1696 (C=O) cm^−1^. Mp: 225–226 °C. ^1^H and ^13^C NMR data are consistent with those reported in the literature [111].

#### 3.4.4. 5-Chloro-3-oxo-1λ^3^-benzo[d][1,2]iodaoxol-1(3H)-yl acetate (**7c**)

^1^H NMR (500 MHz, CDCl_3_): δ 2.27 (3H, s), 7.87 (1H, dd, *J* = 8.6, 2.3 Hz), 7.93 (1H, d, *J* = 9.2 Hz), 8.22 (1H, d, *J* = 1.7 Hz) ppm. ^13^C NMR (125 MHz, CDCl_3_): δ 20.2, 115.5, 130.4, 130.9, 133.0, 136.0, 138.8, 166.7, 176.4 ppm. IR (ATR, KBr): *ν* 1698 (C=O) cm^−1^. Mp: 244–245 °C. ^1^H and ^13^C NMR data are consistent with those reported in the literature [105].

#### 3.4.5. 5-Bromo-3-oxo-1λ^3^-benzo[d][1,2]iodaoxol-1(3H)-yl acetate (**7d**)

^1^H NMR (500 MHz, CDCl_3_): δ 2.27 (3H, s), 7.85 (1H, d, *J* = 9.2 Hz), 8.01 (1H, dd, *J* = 8.6, 2.3 Hz), 8.37 (1H, d, *J* = 1.8 Hz) ppm. ^13^C NMR (125 MHz, CDCl_3_): δ 20.2, 116.5, 126.6, 130.7, 131.0, 136.1, 138.9, 166.6, 176.4 ppm. IR (ATR, KBr): *ν* 1680 (C=O) cm^−1^. Mp: 226–228 °C. ^1^H and ^13^C NMR data are consistent with those reported in the literature [112].

#### 3.4.6. 5-Methyl-3-oxo-1λ^3^-benzo[d][1,2]iodaoxol-1(3H)-yl acetate (**7e**)

^1^H NMR (500 MHz, CDCl_3_): δ 2.25 (3H, s), 2.56 (3H, s), 7.73 (1H, dd, *J* = 8.6, 1.7 Hz), 7.84 (1H, d, *J* = 8.6 Hz), 8.07 (1H, d, *J* = 1.8 Hz) ppm. ^13^C NMR (125 MHz, CDCl_3_): δ 20.3, 20.8, 114.6, 128.9, 133.6, 137.1, 142.3, 168.3, 176.4 ppm. IR (ATR, KBr): *ν* 1682 (C=O), 1659 (C=O) cm^−1^. Mp: 215–217 °C. ^1^H and ^13^C NMR data are consistent with those reported in the literature [105].

#### 3.4.7. 5-Methoxy-3-oxo-1λ^3^-benzo[d][1,2]iodaoxol-1(3H)-yl acetate (**7f**)

^1^H NMR (500 MHz, CDCl_3_): δ 2.25 (3H, s), 3.94 (3H, s), 7.46 (1H, dd, *J* = 9.2, 2.9 Hz), 7.74 (1H, d, *J* = 2.9 Hz), 7.81 (1H, d, *J* = 8.6 Hz) ppm. ^13^C NMR (125 MHz, CDCl_3_): δ 20.3, 56.2, 106.8, 115.9, 124.5, 129.7, 130.6, 162.7, 168.1, 176.4 ppm. IR (ATR, KBr): *ν* 1697 (C=O), 1681 (C=O), 1656 (C=O) cm^−1^. Mp: 207–209 °C. ^1^H and ^13^C NMR data are consistent with those reported in the literature [105].

#### 3.4.8. 3-Oxo-1λ^3^-benzo[d][1,2]iodaoxole-1,5(3H)-diyl diacetate (**7g**)

^1^H NMR (500 MHz, CDCl_3_): δ 2.26 (3H, s), 2.37 (3H, s), 7.68 (1H, dd, *J* = 8.9, 2.6 Hz), 7.96–8.02 (2H, m) ppm. ^13^C NMR (125 MHz, CDCl_3_): δ 20.3, 21.1, 113.6, 126.2, 129.8, 130.3, 130.9, 153.6, 167.2, 168.7, 176.5 ppm. IR (ATR, KBr): *ν* 1690 (C=O) cm^−1^. Mp: 153–154 °C. HRMS (DART, m/z) calcd for C_11_H_10_IO_6_ [M + H]^+^: 364.9517; found: 364.9518.

#### 3.4.9. 3-Oxo-5-(trifluoromethyl)-1λ^3^-benzo[d][1,2]iodaoxol-1(3H)-yl acetate (**7h**)

^1^H NMR (500 MHz, CDCl_3_): δ 2.29 (3H, s), 8.14 (1H, dd, *J* = 8.6, 1.7 Hz), 8.20 (1H, d, *J* = 8.6 Hz), 8.52 (1H, d, *J* = 1.7 Hz) ppm. ^13^C NMR (125 MHz, CDCl_3_): δ 20.2, 121.9, 122.8 (q, *J* = 271.4 Hz), 130.1 (d, *J* = 3.6 Hz), 130.4 (d, *J* = 9.5 Hz), 132.4 (d, *J* = 2.4 Hz), 134.5 (q, *J* = 33.8 Hz), 166.7, 176.5 ppm. ^19^F NMR (470 MHz, CDCl_3_): δ −64.9 ppm. IR (ATR, KBr): *ν* 1692 (C=O), 1647 (C=O) cm^−1^. Mp: 212–213 °C. HRMS (DART, m/z) calcd for C_10_H_7_F_3_IO_4_ [M + H]^+^: 374.9336; found: 374.9334.

#### 3.4.10. 5-Nitro-3-oxo-1λ^3^-benzo[d][1,2]iodaoxol-1(3H)-yl acetate (**7i**)

^1^H NMR (500 MHz, CDCl_3_): δ 2.30 (3H, s), 8.27 (1H, d, *J* = 9.2 Hz), 8.71 (1H, dd, *J* = 9.0, 2.5 Hz), 9.04 (1H, d, *J* = 2.3 Hz) ppm. ^13^C NMR (125 MHz, CDCl_3_): δ 20.2, 124.1, 127.6, 129.8, 131.0, 131.5,150.8, 165.7, 176.6 ppm. IR (ATR, KBr): *ν* 1705 (C=O), 1665 (C=O) cm^−1^. Mp: 209–210 °C. ^1^H and ^13^C NMR data are consistent with those reported in the literature [107].

## 4. Conclusions

We have presented a practical synthetic method for IBA from 2-IB without contamination by hazardous pentavalent IBX using cost-effective Oxone^®^ in aqueous solution. This highly safe, convenient method operates under mild conditions such as room temperature, which contrasts with traditional method using reflux conditions and expensive NaIO_4_ in AcOH solution. The use of mild conditions circumvents the problem of the formation of byproducts such as potentially explosive pentavalent cyclic hypervalent iodine compound, i.e., IBX; the contamination of IBX into IBA is generally not desired for safety reasons. The reaction time can be shortened by heating; in this case, addition of a ferric salt in our reaction system can effectively suppress the formation of IBX as byproducts. In addition, a convenient derivatization of the hydroxy group of IBAs by solvolytic treatment is presented. These derivatizations were generally achieved under mild conditions below 80 °C. Our methods, which do not require any chromatography technique, can be performed safely and would be suitable for large-scale synthesis.

## Data Availability

The data presented in this study are available in the communication.

## Supplementary Materials

Supplementary materials are available online, ^1^H NMR spectroscopic data for the compounds **2a**–**v** and **4**.

## Sample Availability

Samples of the compounds are not available from the authors.
