# Supplementary material for: Practical Synthesis of 2-Iodosobenzoic Acid (IBA) without Contamination by Hazardous 2-Iodoxybenzoic Acid (IBX) under Mild Conditions
Source: Molecules. 2021 Mar 27;26(7):1897. doi: 10.3390/molecules26071897 (PMC8036297; doi:10.3390/molecules26071897)

Practical Synthesis of 2-Iodosobenzoic Acids (IBAs) without Contamintion by Hazardous 2-Iodoxybenzoic Acids (IBXs) under Mild Conditions

Hideyasu China[1, 2*], Nami Kageyama[2], Hodaka Yatabe[2], Naoto Takenaga[3], and Toshifumi Dohi[2,*]

[1] Department of Medical Bioscience, Nagahama Institute of Bio-Science and Technology, 1266, Tamuracho Nagahama-shi, Shiga, 526-0829, Japan

[2] College of Pharmaceutical Sciences, Ritsumeikan University, 1-1-1 Nojihigashi, Kusatsu, Shiga 525–8577, Japan

[3] Faculty of Pharmacy, Meijo University, 150 Yagotoyama, Tempaku-ku, Nagoya 468-8503, Japan

^{1}H NMR Spectrum (400 MHz, DMSO-*d6*) of **2a**

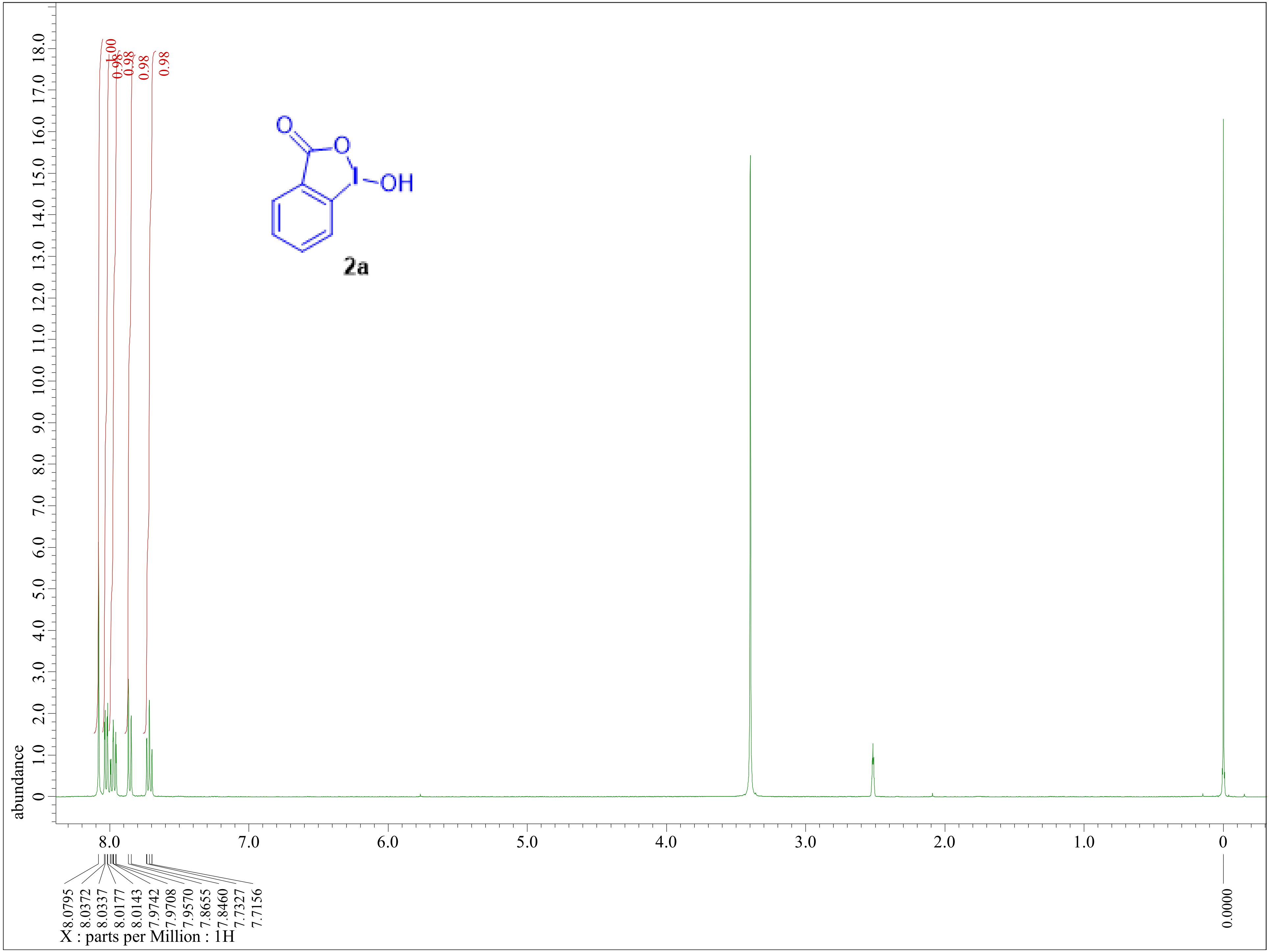

^{1}H NMR Spectrum (500 MHz, DMSO-*d6*) of **2b**

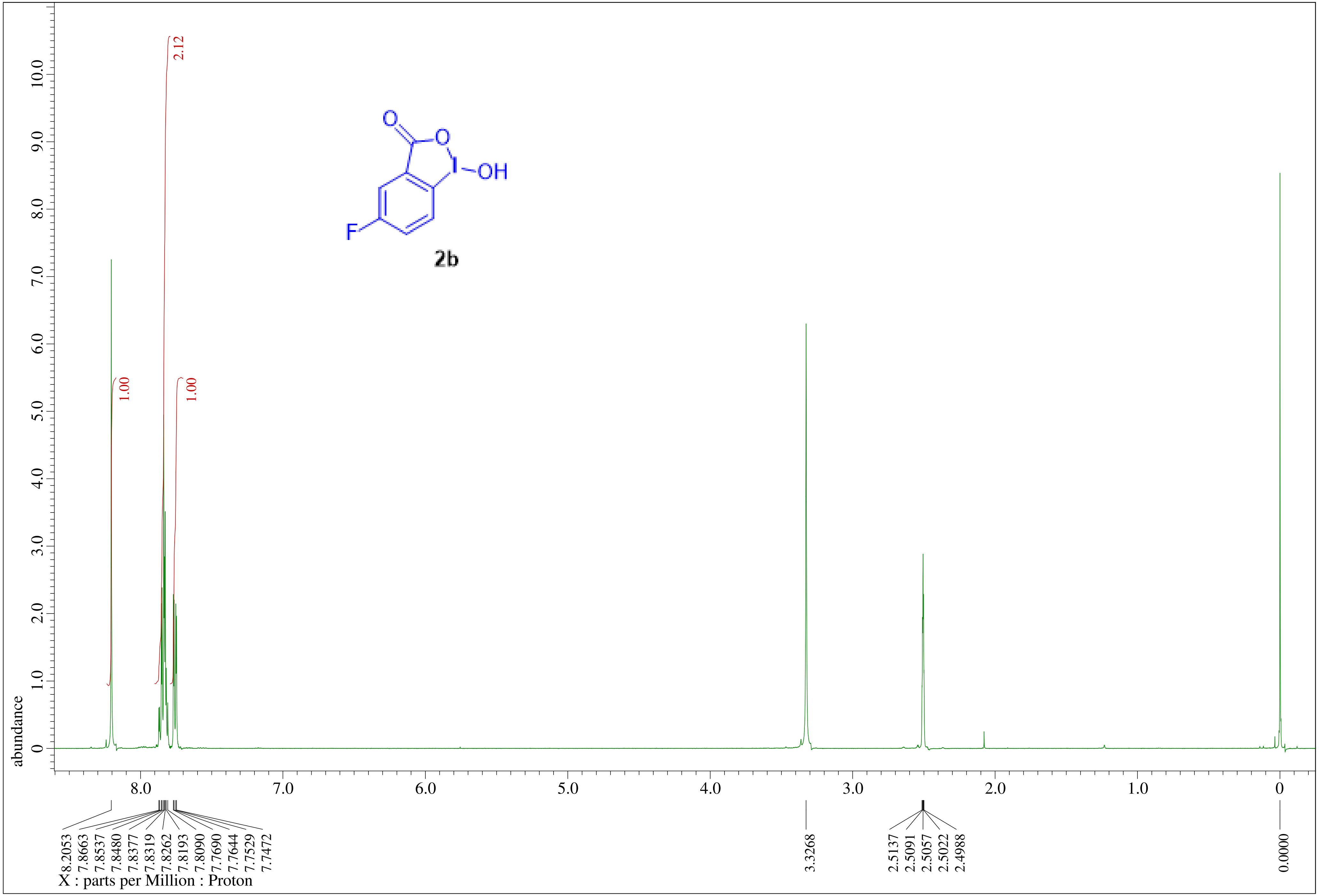

^1H NMR Spectrum (400 MHz, DMSO-*d6*) of **2c**

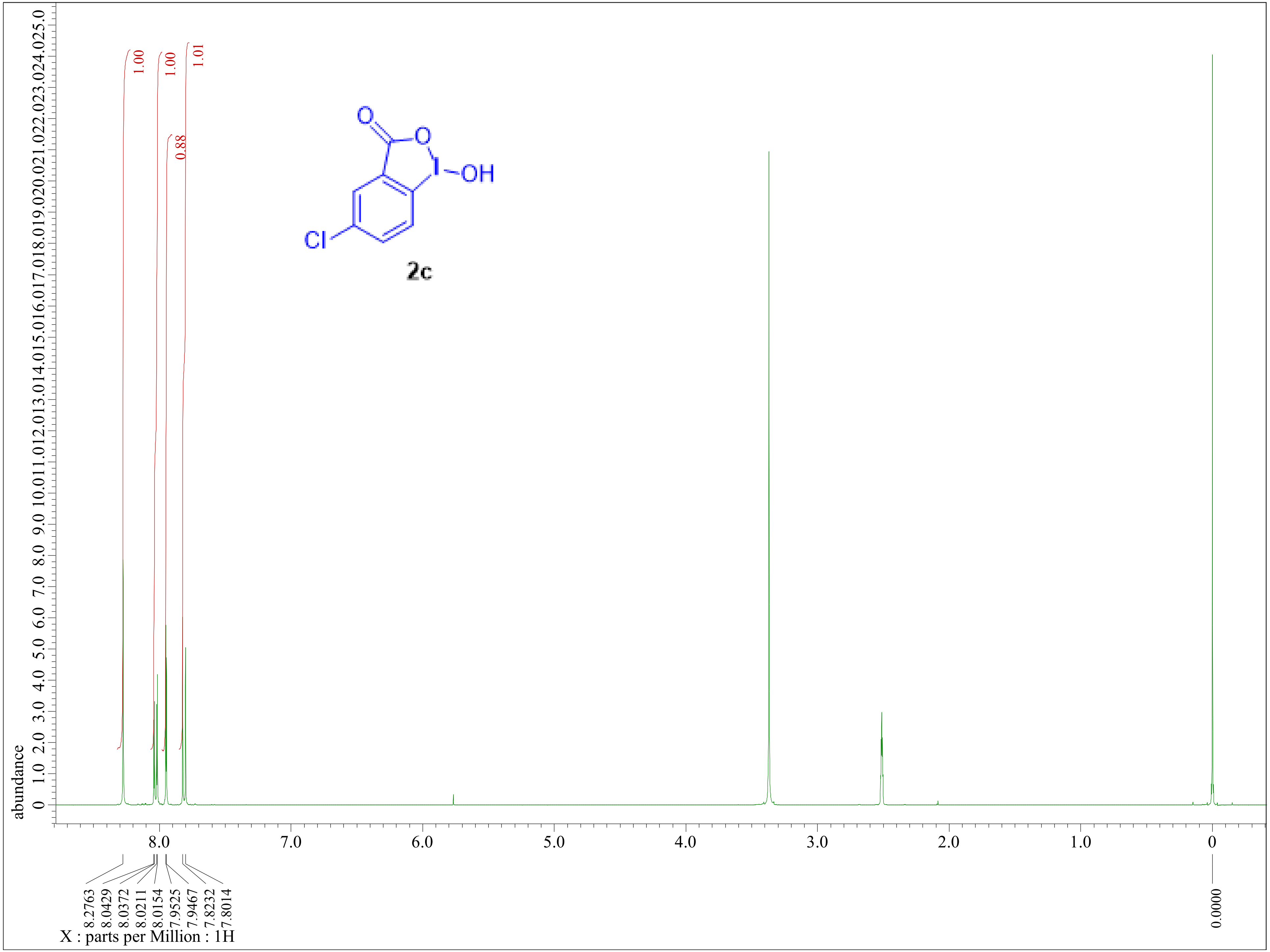

^{1}H NMR Spectrum (400 MHz, DMSO-*d6*) of **2d**

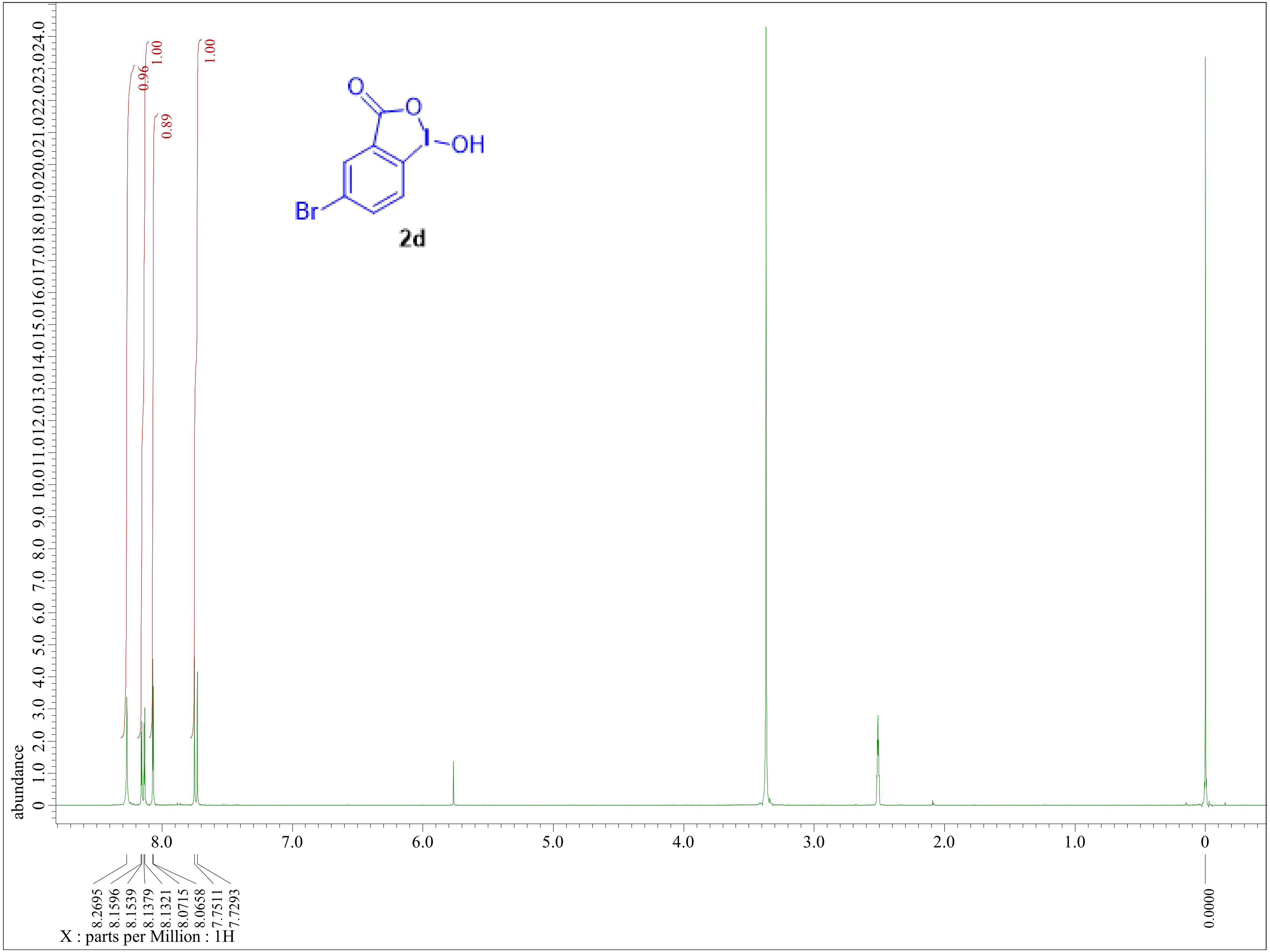

^{1}H NMR Spectrum (400 MHz, DMSO-*d6*) of **2e**

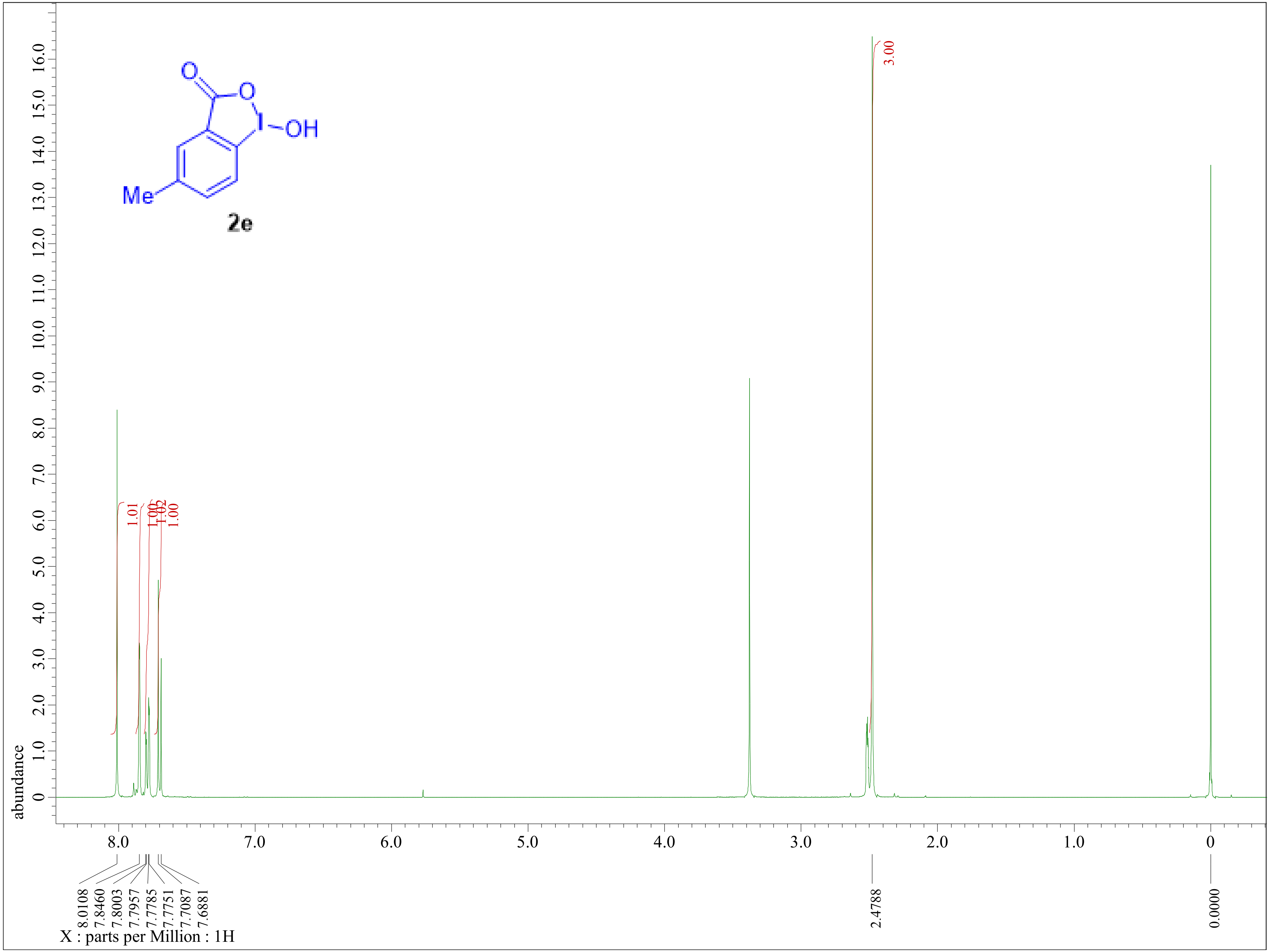

^{1}H NMR Spectrum (400 MHz, DMSO-*d6*) of **2f**

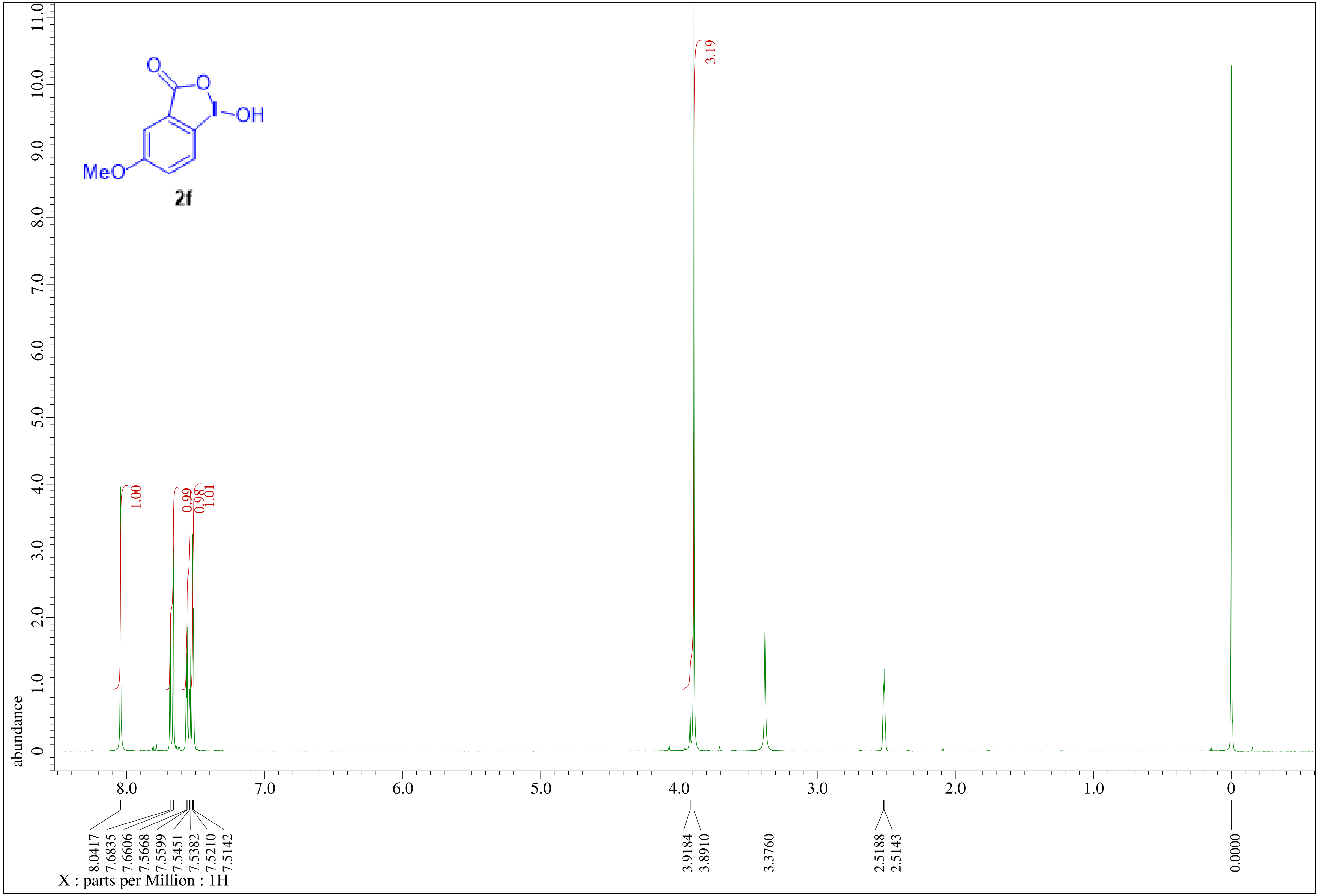

^{1}H NMR Spectrum (500 MHz, DMSO-*d6*) of **2g**

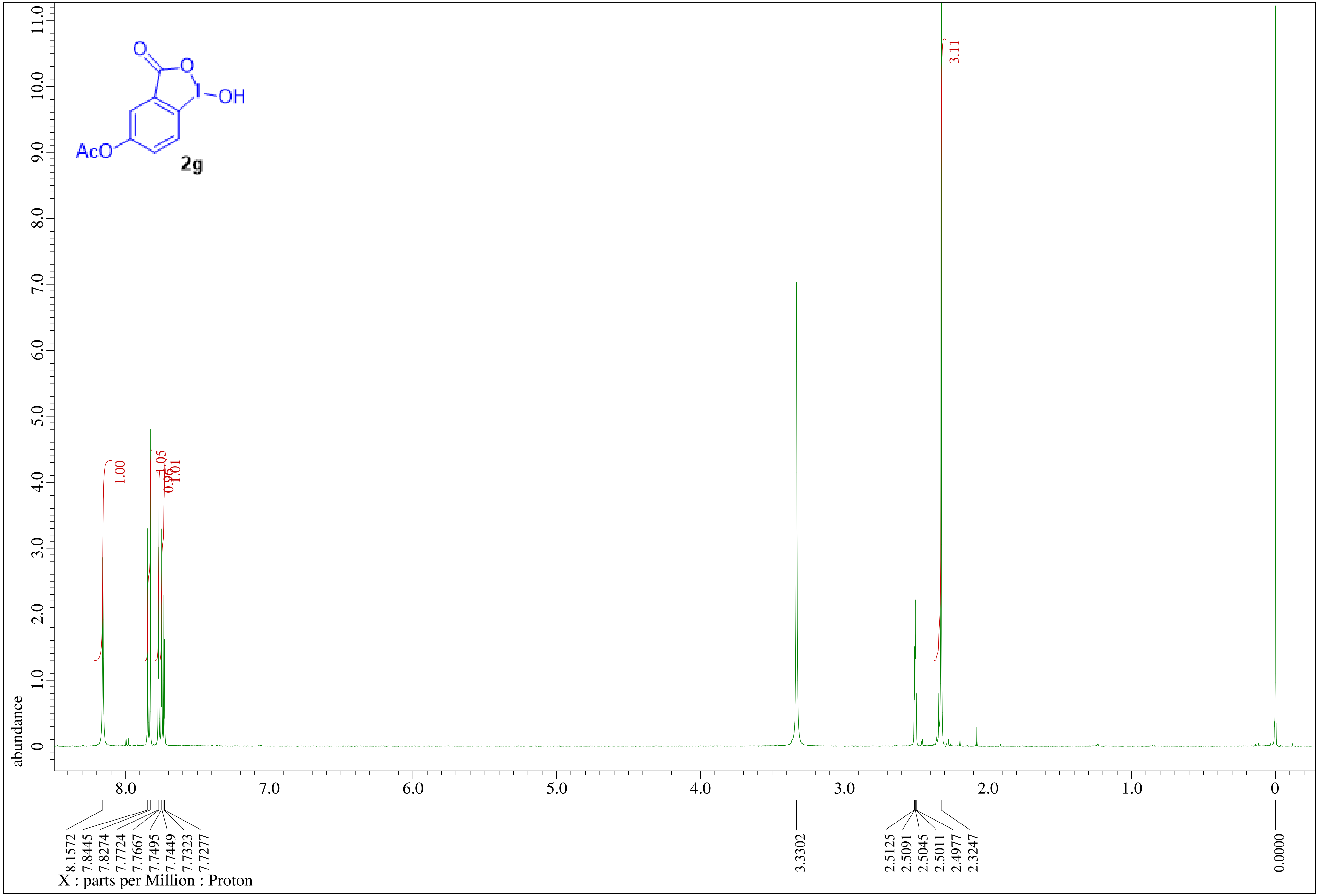

^{1}H NMR Spectrum (500 MHz, DMSO-*d6*) of **2h**

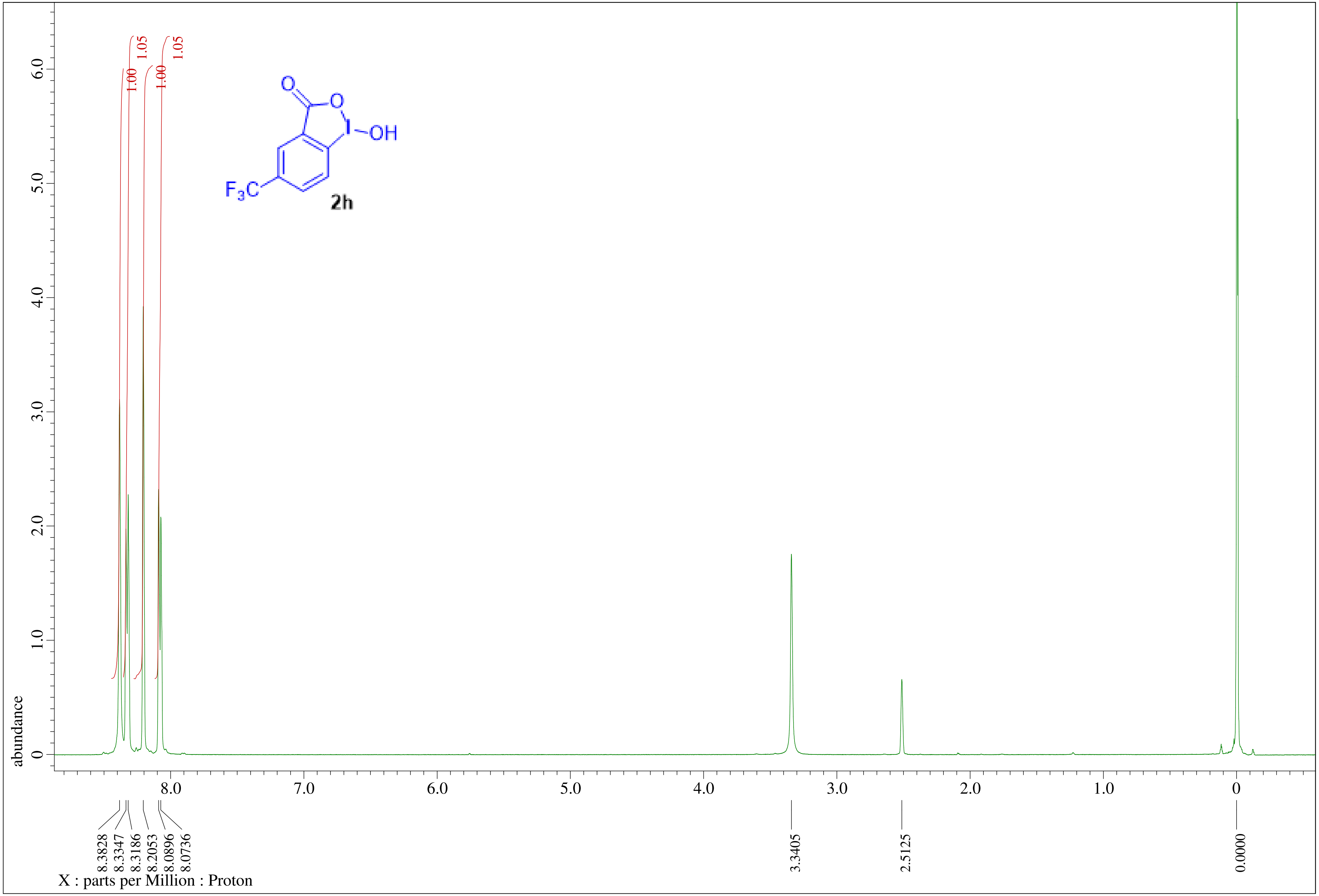

^{1}H NMR Spectrum (400 MHz, DMSO-*d6*) of **2i**

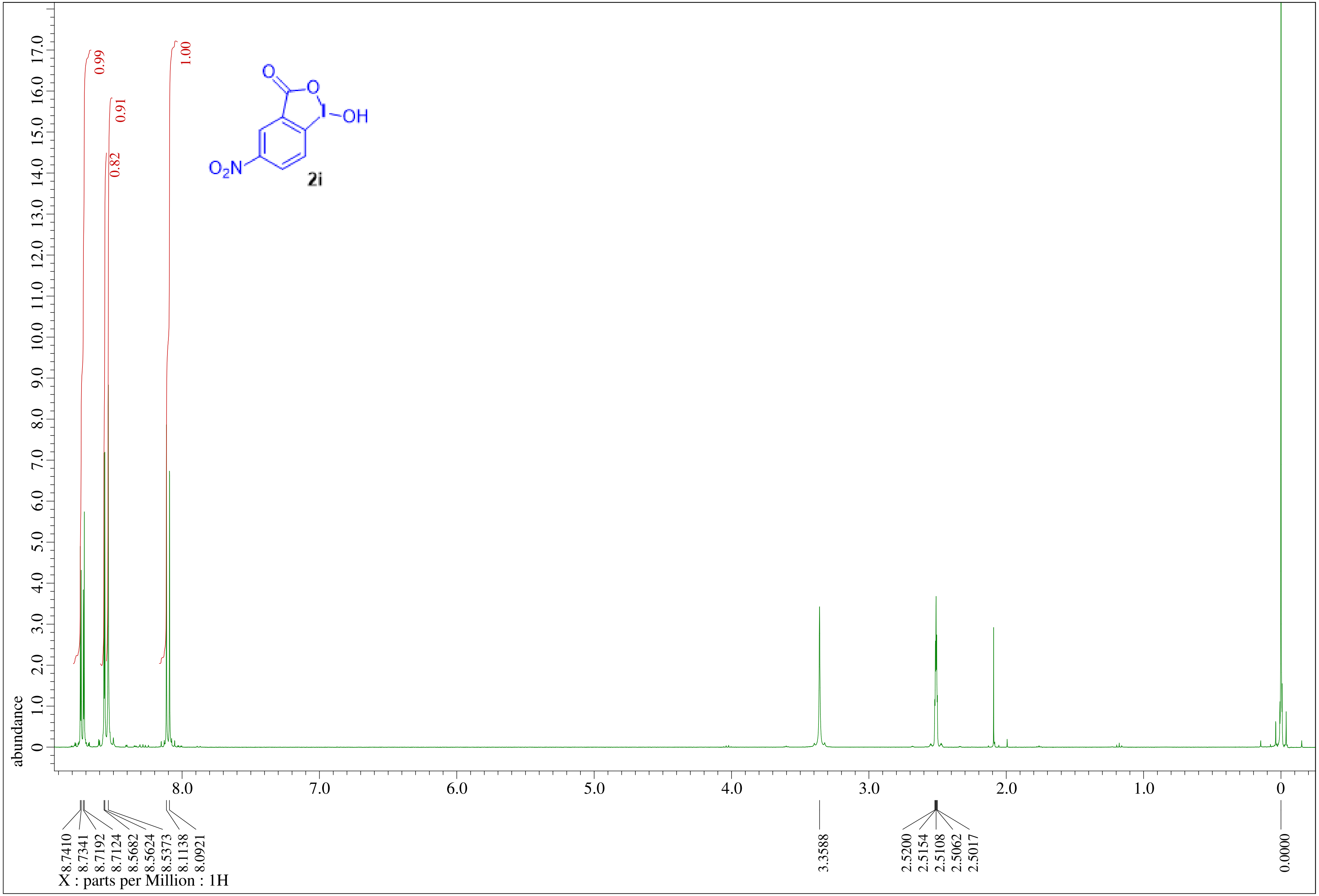

^{1}H NMR Spectrum (500 MHz, DMSO-*d6*) of **2j**

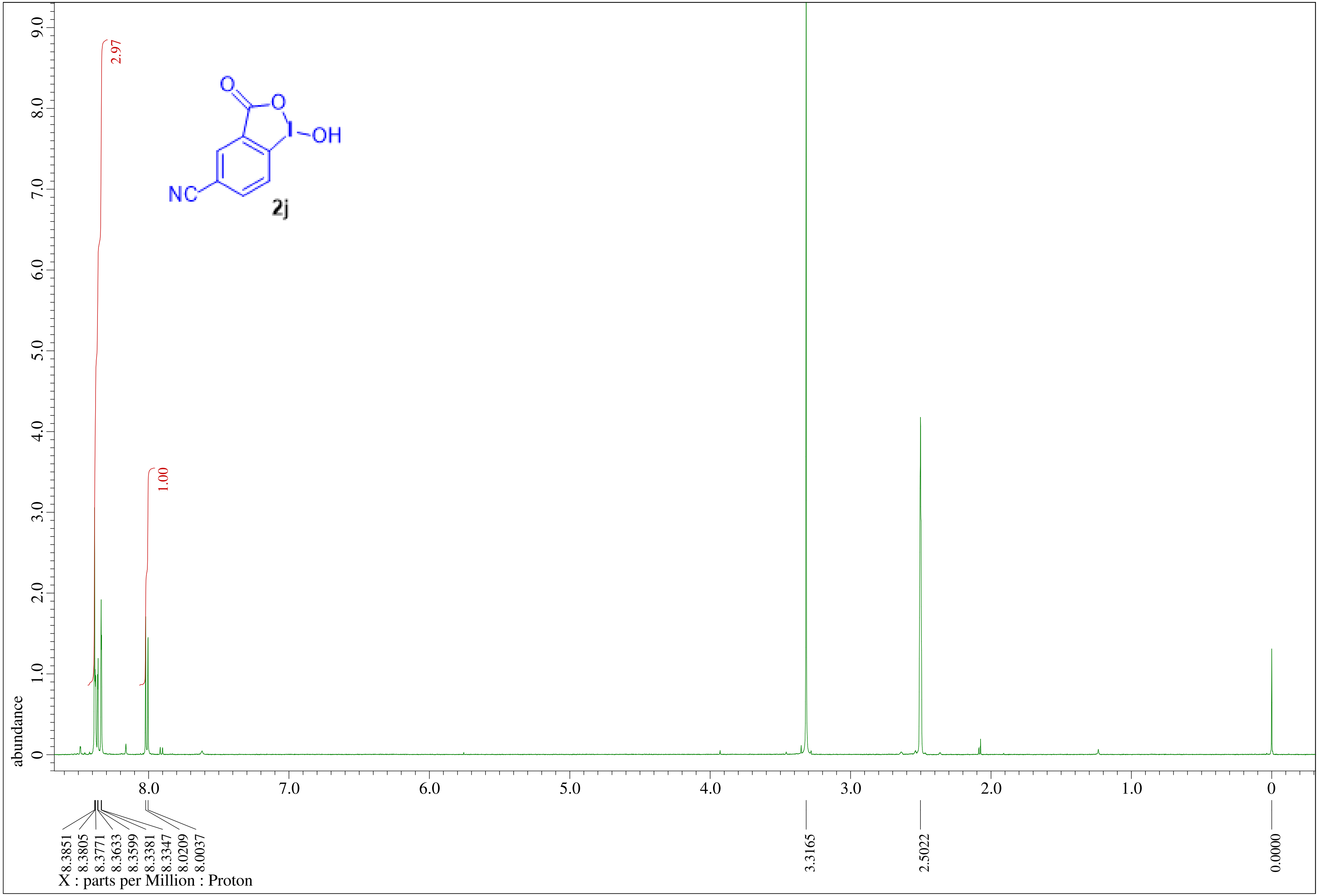

^{1}H NMR Spectrum (400 MHz, DMSO-*d6*) of **2k**

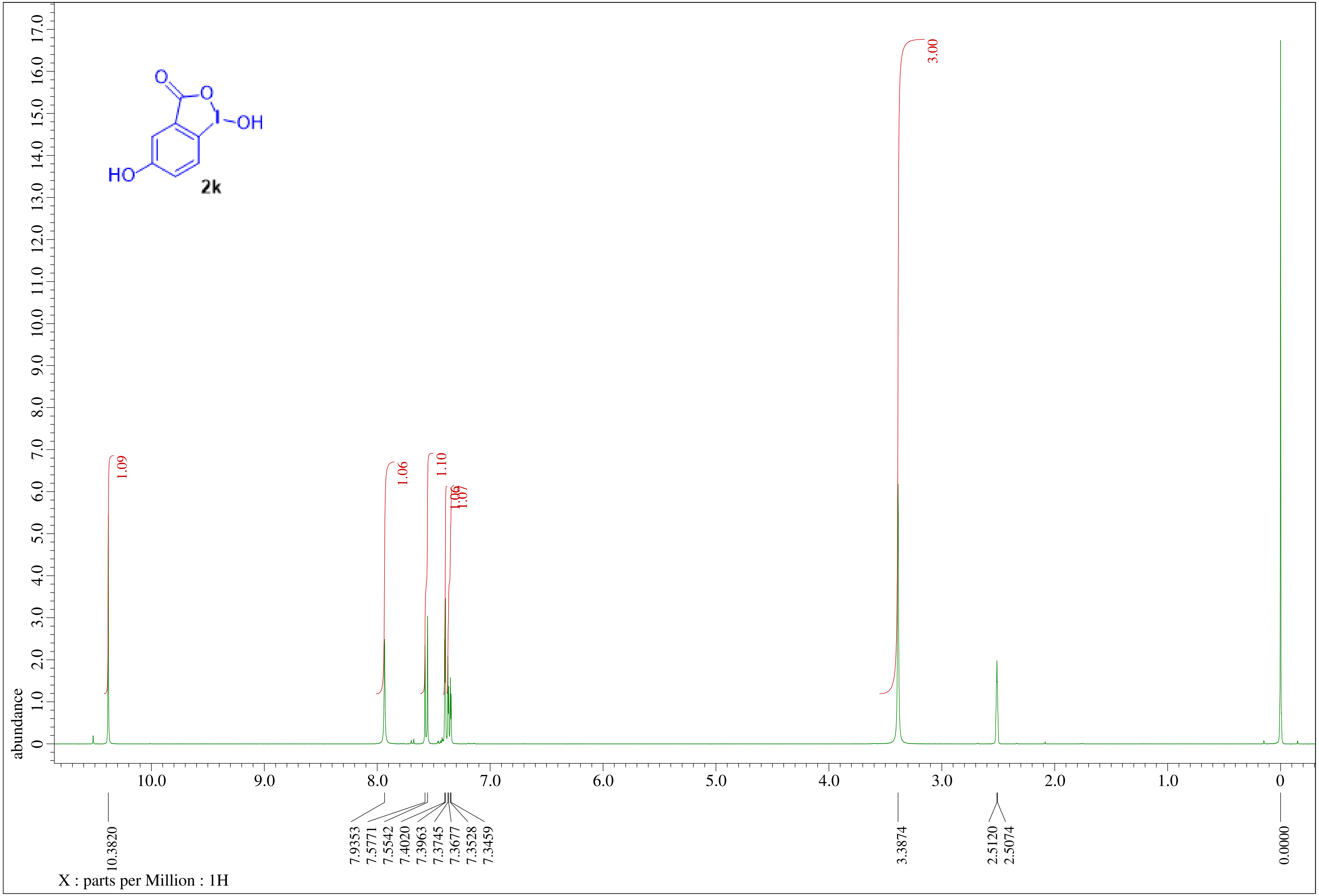

^{1}H NMR Spectrum (500 MHz, DMSO-*d6*) of **2l**

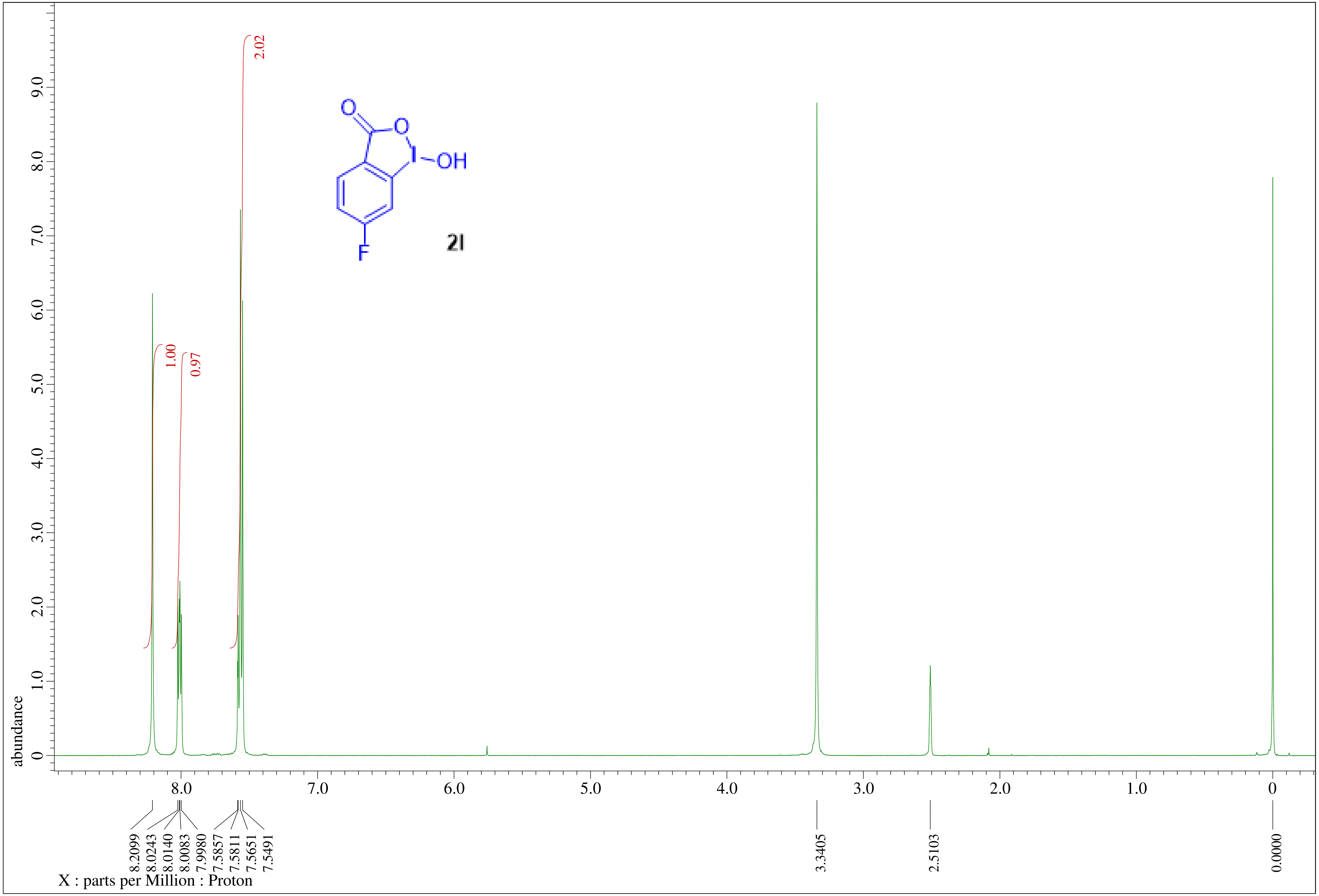

^{1}H NMR Spectrum (400 MHz, DMSO-*d6*) of **2m**

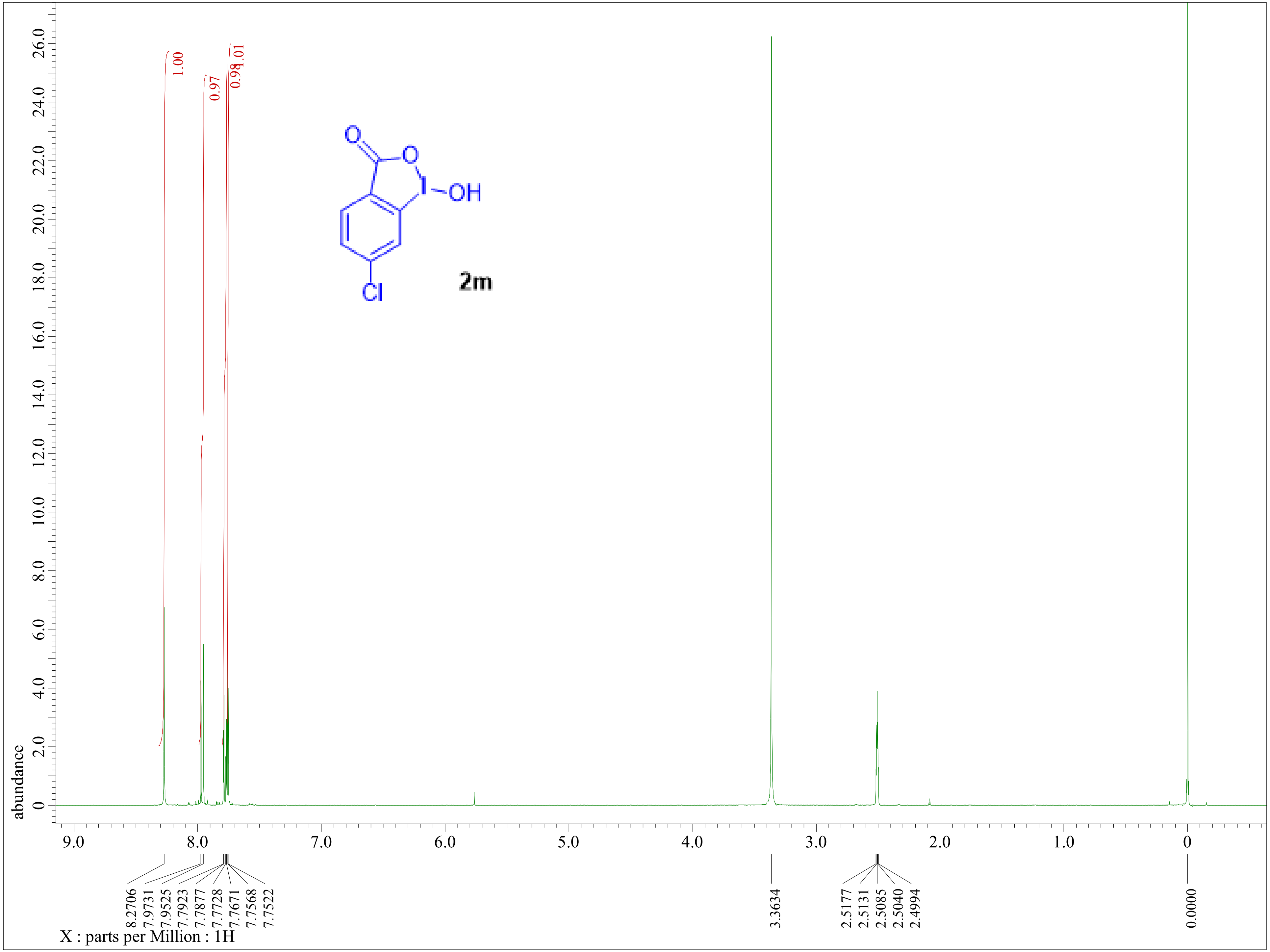

^{1}H NMR Spectrum (500 MHz, DMSO-*d6*) of **2n**

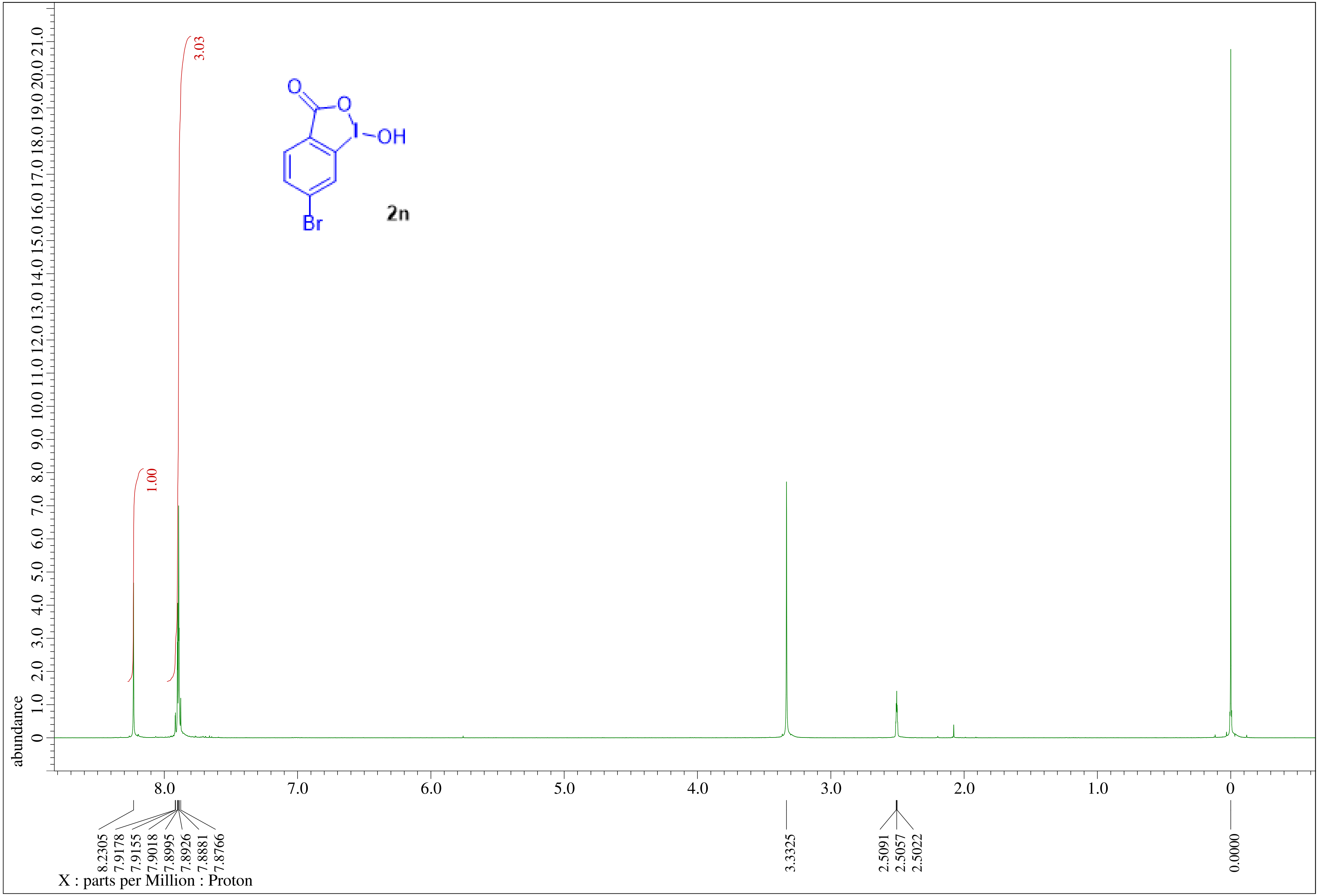

^{1}H NMR Spectrum (400 MHz, DMSO-*d6*) of **2o**

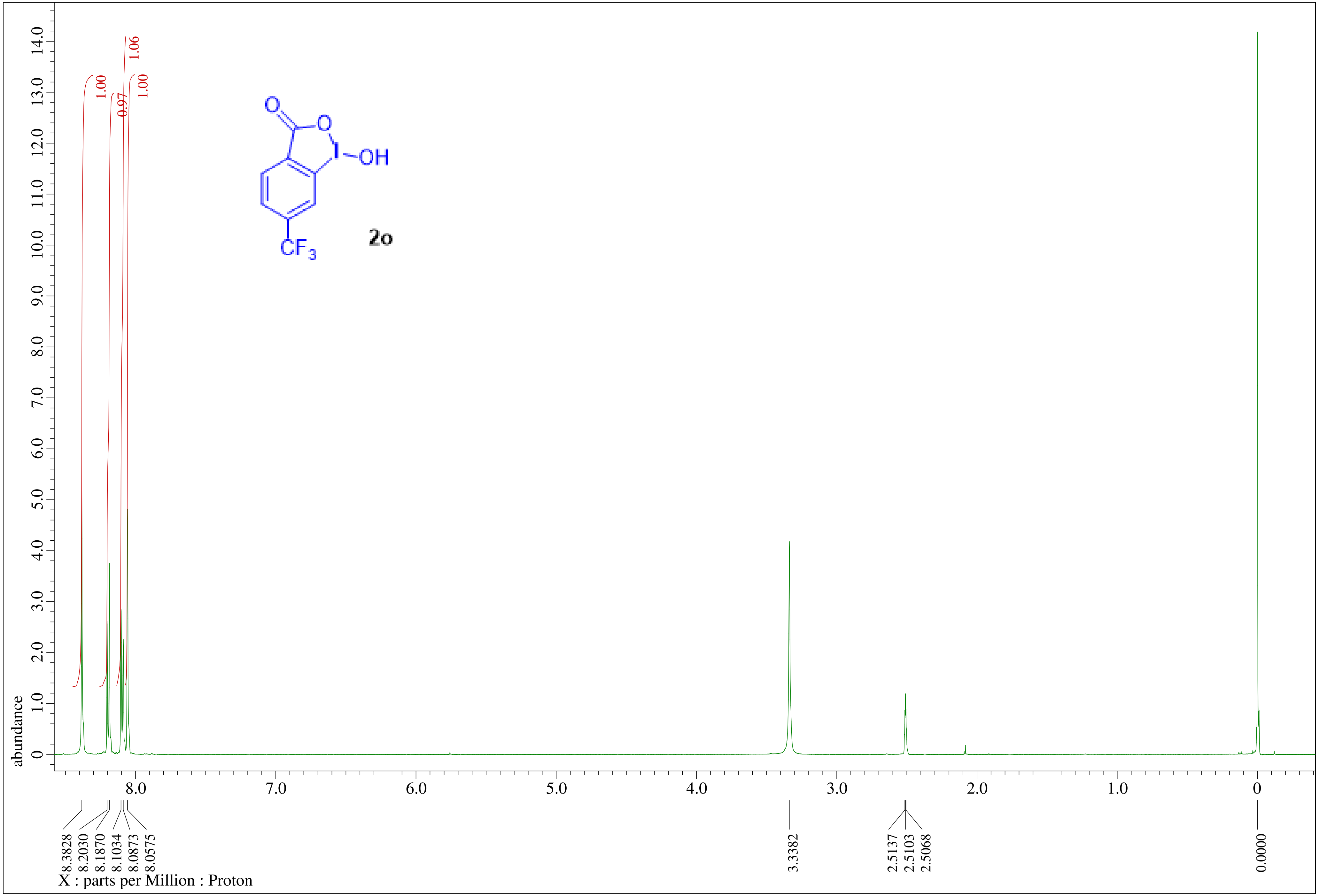

^{1}H NMR Spectrum (500 MHz, DMSO-*d6*) of **2p**

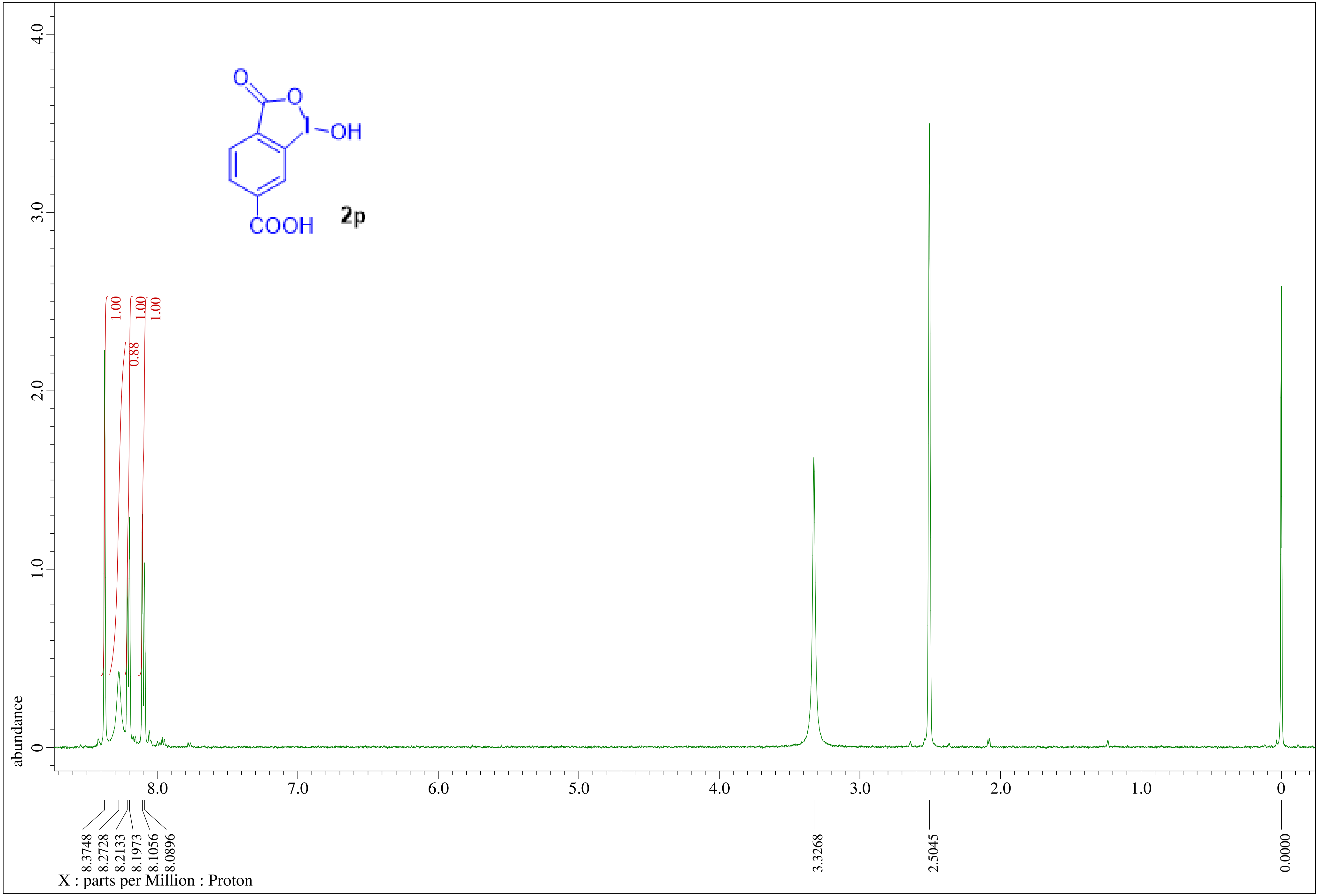

^{1}H NMR Spectrum (500 MHz, DMSO-*d6*) of **2q**

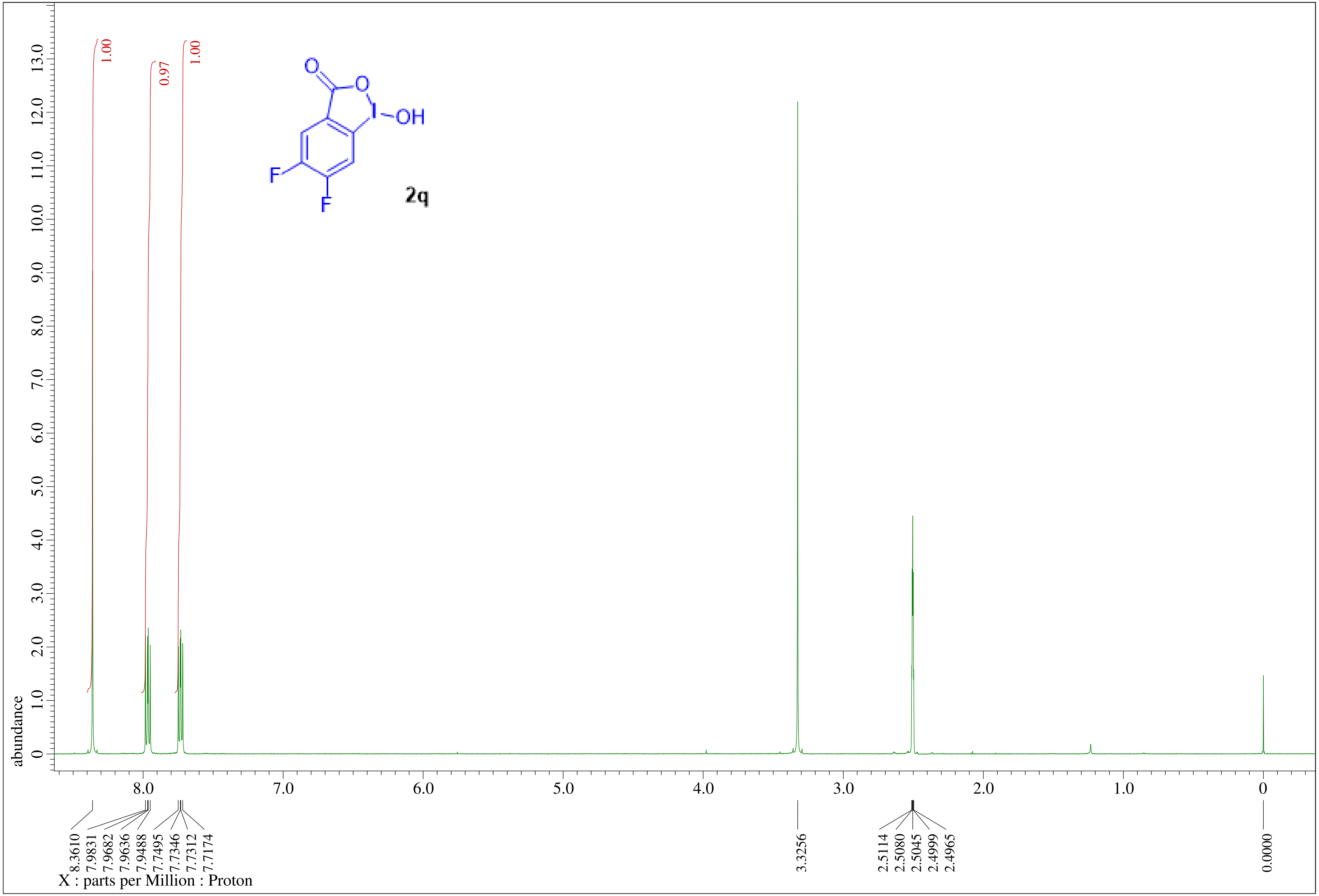

^{1}H NMR Spectrum (500 MHz, DMSO-*d6*) of **2r**

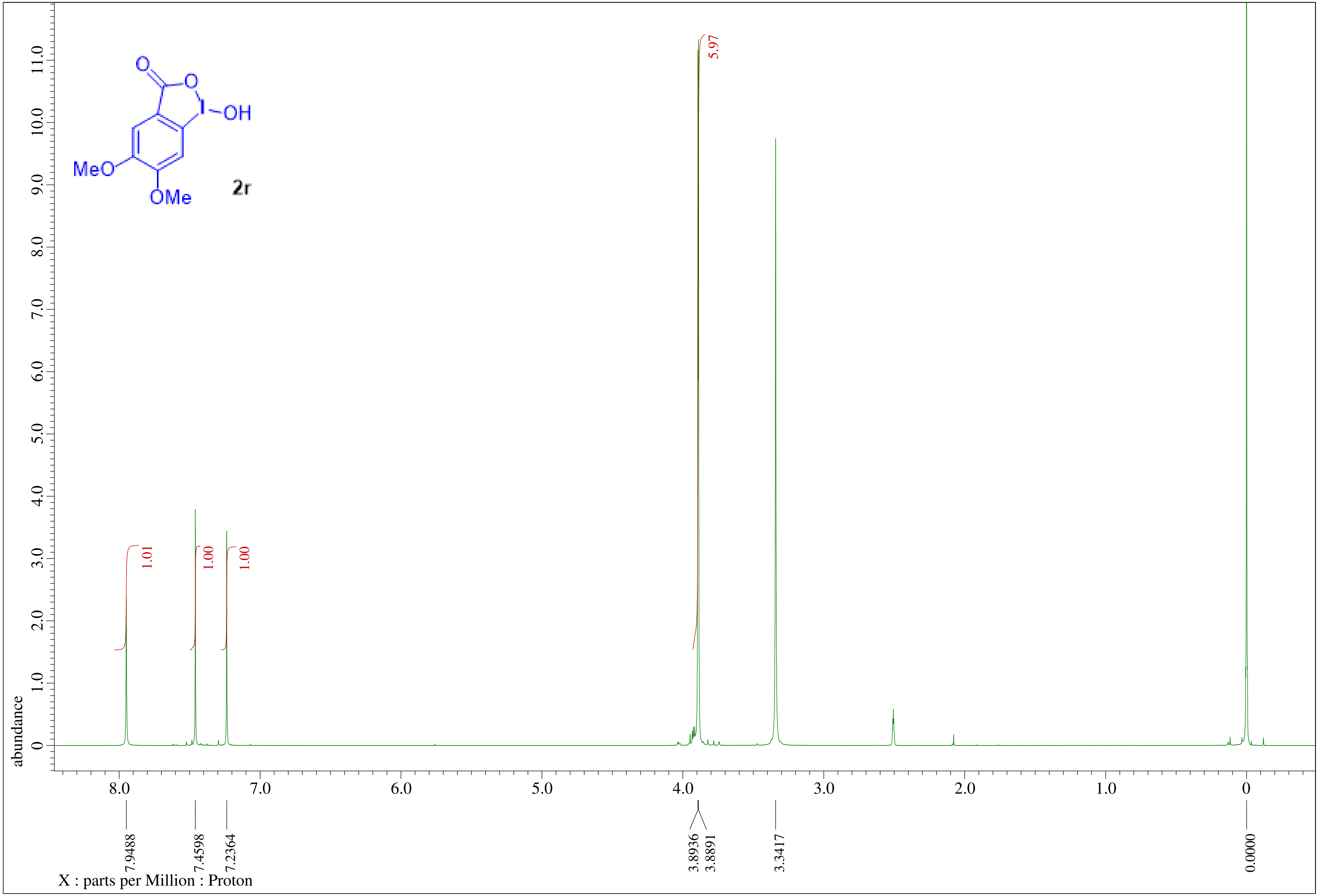

^{1}H NMR Spectrum (500 MHz, DMSO-*d6*) of **2s**

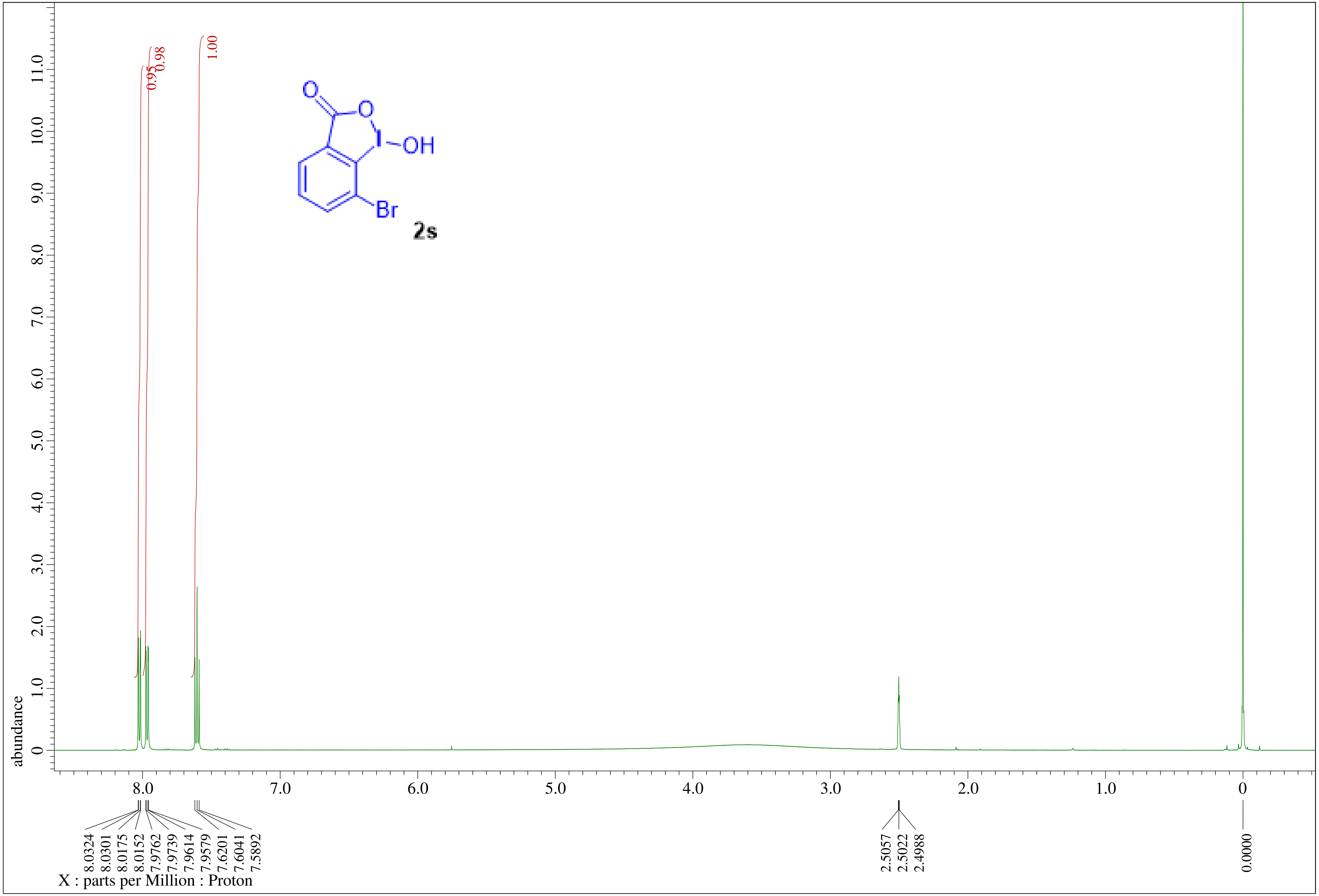

^{1}H NMR Spectrum (400 MHz, DMSO-*d6*) of **2t**

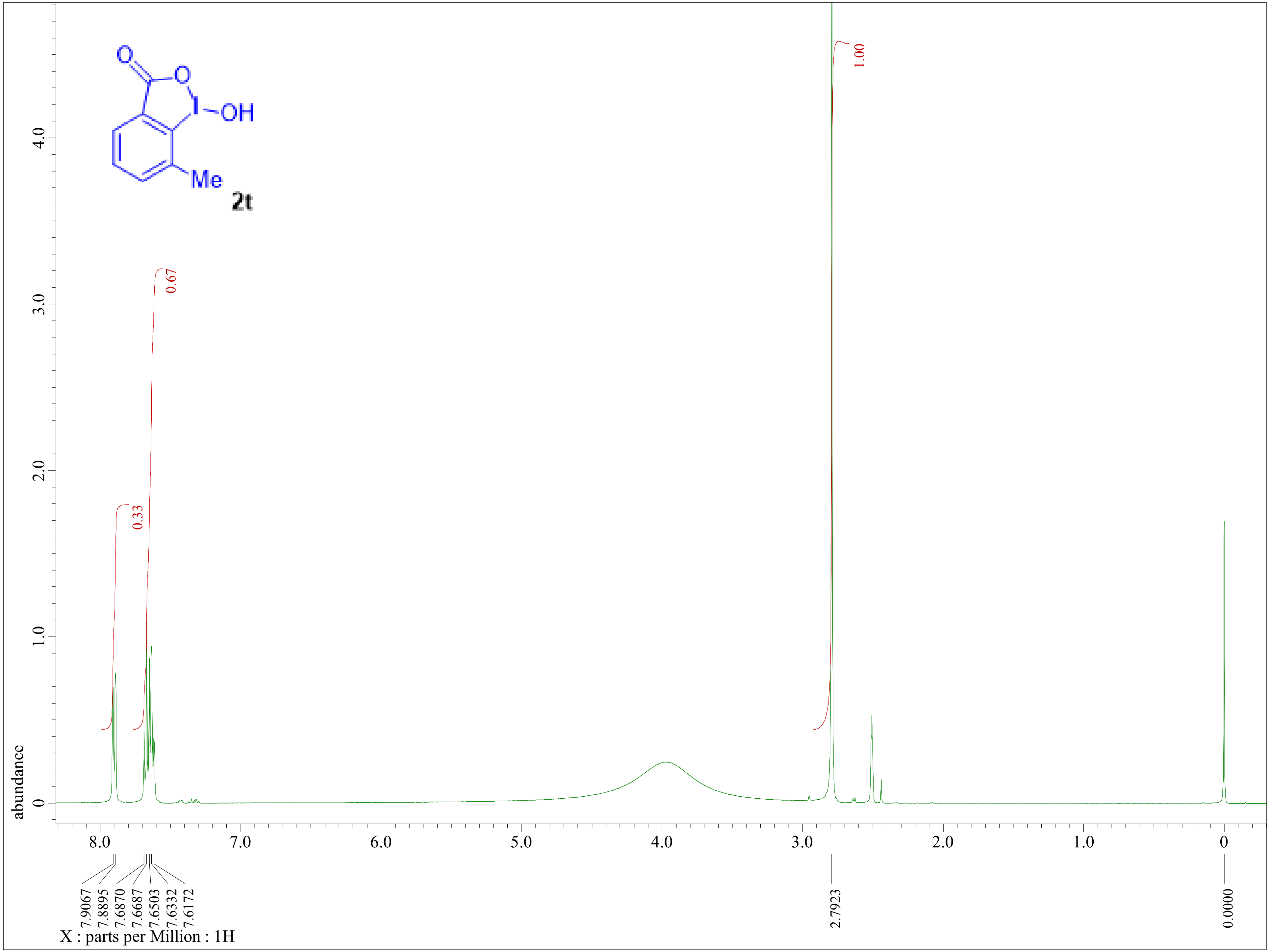

^{1}H NMR Spectrum (400 MHz, DMSO-*d6*) of **2u**

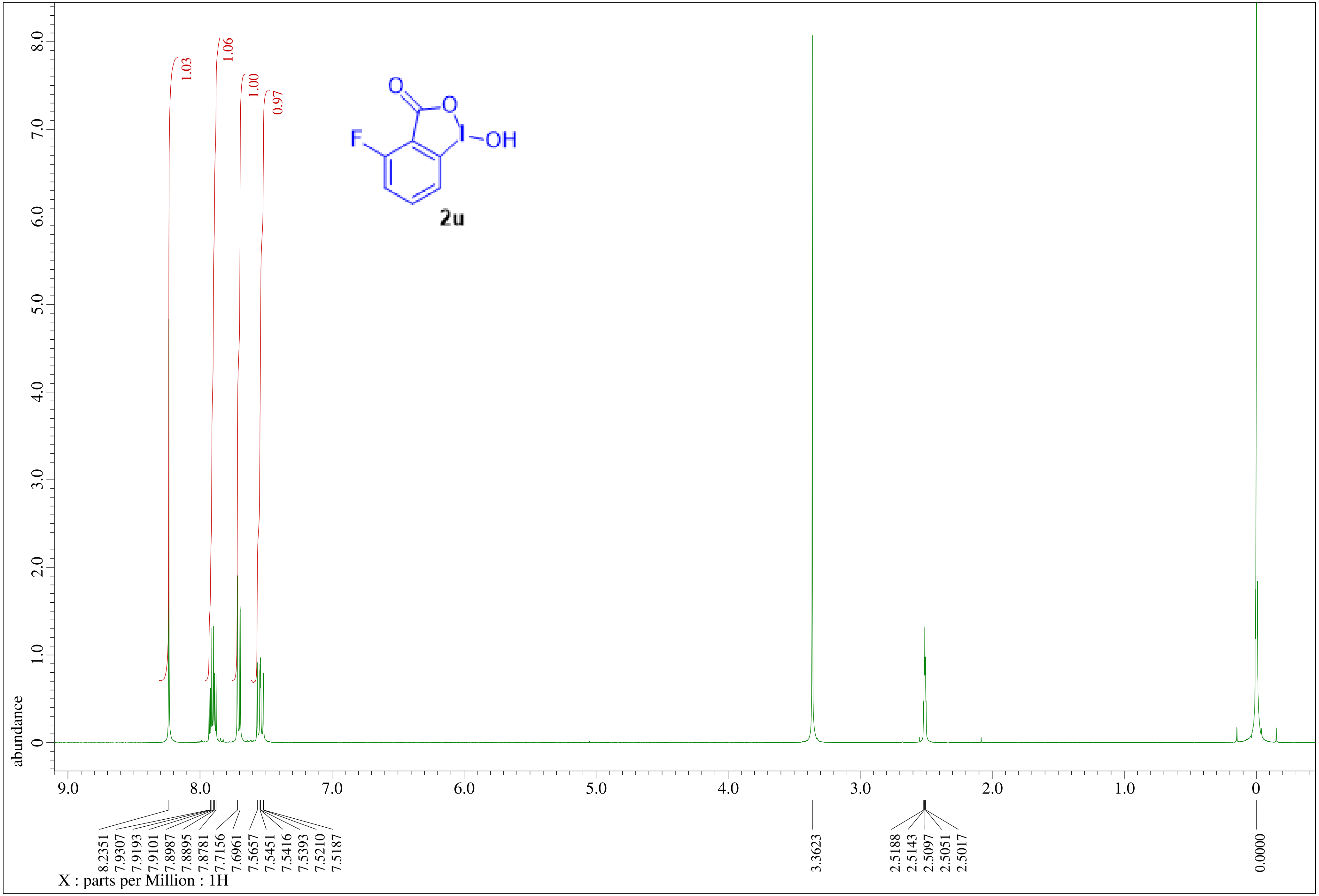

^1H NMR Spectrum (400 MHz, DMSO-*d6*) of **2v**

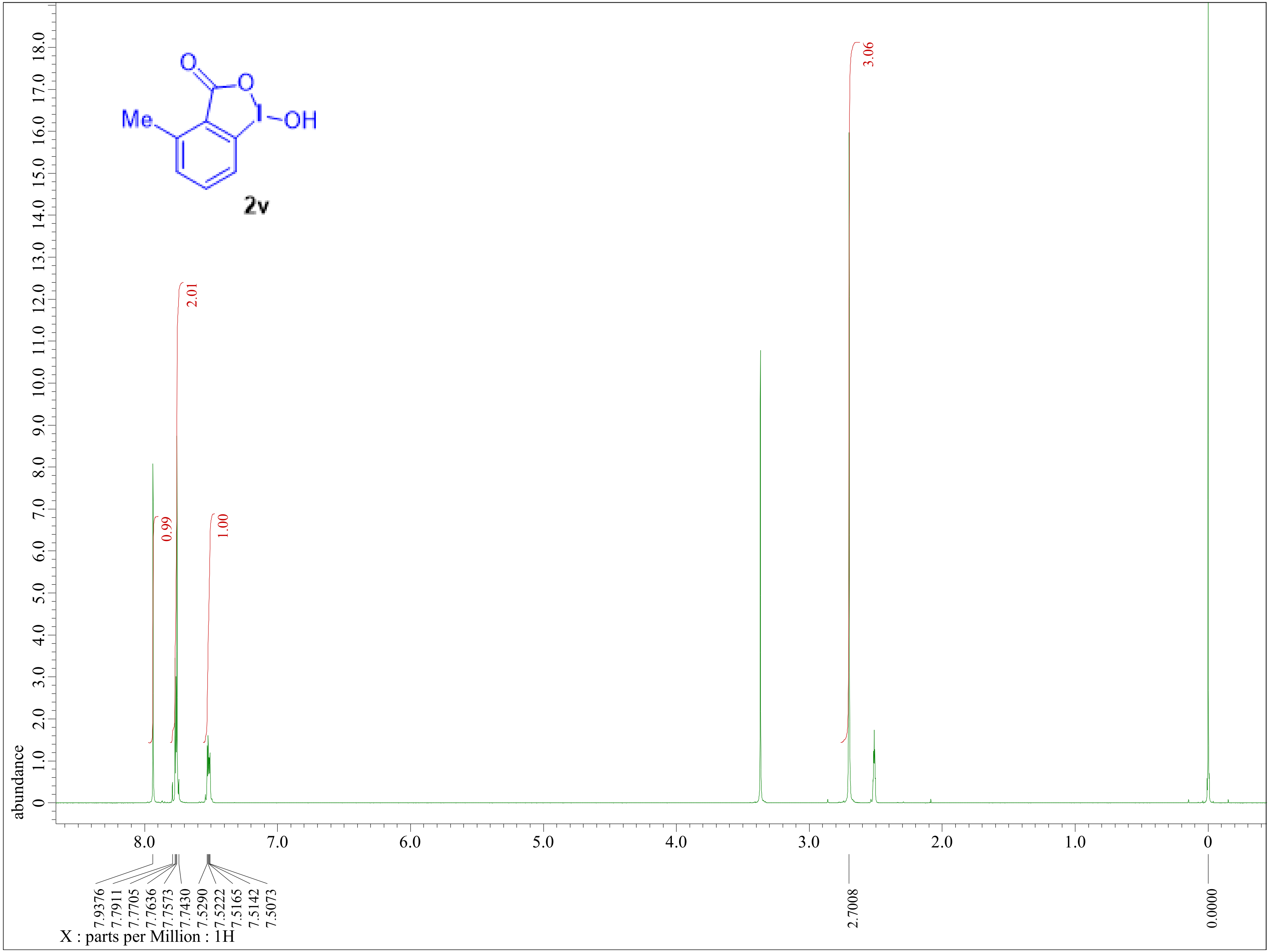

^{1}H NMR Spectrum (400 MHz, DMSO-*d6*) of 4

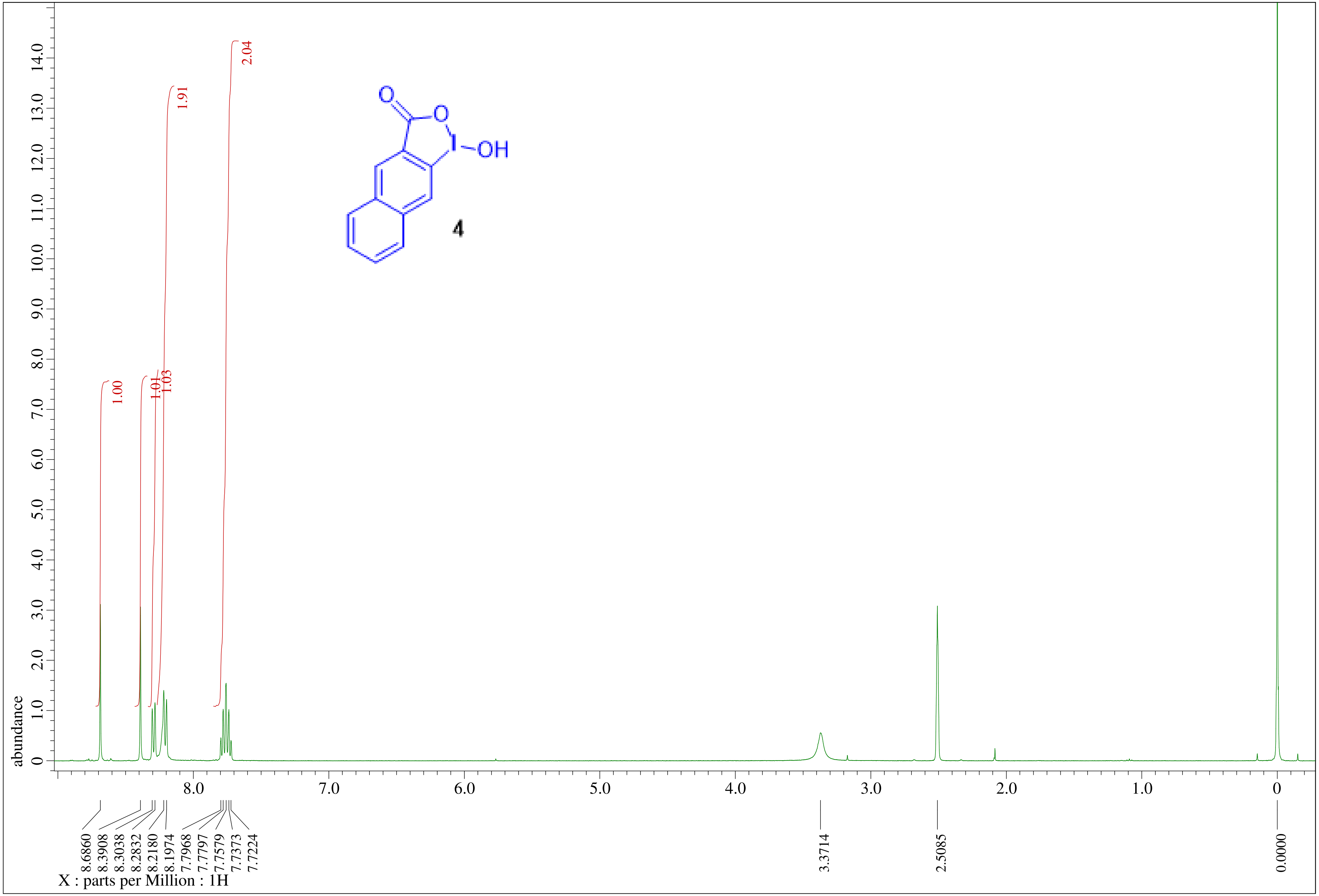

Supplement: Supplementary file 1 [file molecules-26-01897-s001.pdf]
